# Cryo-EM reveals the structural basis of microtubule depolymerization by kinesin-13s

**DOI:** 10.1038/s41467-018-04044-8

**Published:** 2018-04-25

**Authors:** Matthieu P.M.H. Benoit, Ana B. Asenjo, Hernando Sosa

**Affiliations:** Department of Physiology and Biophysics, Albert Einstein College of Medicine, Bronx, NY 10461 USA

## Abstract

Kinesin-13s constitute a distinct group within the kinesin superfamily of motor proteins that promote microtubule depolymerization and lack motile activity. The molecular mechanism by which kinesin-13s depolymerize microtubules and are adapted to perform a seemingly very different activity from other kinesins is still unclear. To address this issue, here we report the near atomic resolution cryo-electron microscopy (cryo-EM) structures of *Drosophila melanogaster* kinesin-13 KLP10A protein constructs bound to curved or straight tubulin in different nucleotide states. These structures show how nucleotide induced conformational changes near the catalytic site are coupled with movement of the kinesin-13-specific loop-2 to induce tubulin curvature leading to microtubule depolymerization. The data highlight a modular structure that allows similar kinesin core motor-domains to be used for different functions, such as motility or microtubule depolymerization.

## Abstract (web summary)

Kinesin-13s are microtubule depolymerases that lack motile activity. Here the authors present the cryo-EM structures of kinesin-13 microtubule complexes in different nucleotide bound states, which reveal how ATP hydrolysis is linked to conformational changes and propose a model for kinesin induced depolymerisation.

## Introduction

Kinesin-13s form a group within the kinesin superfamily of motor proteins that lack motile activity and instead work as microtubule depolymerases^1^. They are important regulators of microtubule dynamics in a variety of eukaryotic cell processes such as mitosis^2–4^, cytokinesis^5^, axonal branching^6^, and ciliogenesis^7^, and are considered promising anti-cancer therapeutic targets^8^.

Like all kinesins, kinesin-13s posses a highly conserved ATPase motor domain that is essential for their function. The motor domain alone is sufficient to induce microtubule depolymerization^9–11^. However, an additional stretch of positively charged residues, N-terminal of the motor called the neck domain, is necessary to achieve an activity similar to the full-length protein^12,13^.

The way that kinesin-13s interact with the microtubule is different from motile kinesins. Motile kinesins couple ATP hydrolysis to unidirectional stepping along the microtubule lattice. Kinesin-13s on the other hand couple ATP hydrolysis to the removal of tubulin subunits at the microtubule ends^14^ and when interacting with the microtubule lattice they undergo directional unbiased one-dimensional diffusion^15^. It is thought that kinesin-13s promote microtubule depolymerization by inducing a curved tubulin configuration incompatible with the microtubule tubular structure^9,10,16^. Kinesin-13 constructs that include the motor domain form complexes with curved tubulin akin to the ones observed at microtubule ends during depolymerization^9–11,17,18^.

Although much is known regarding the mechanism of action of motile kinesins, how a highly conserved motor domain is adapted in kinesin-13s to interact with the microtubule in such a distinct manner, and induce microtubule depolymerization is still not clear. To address these questions here we use cryo-electron microscopy (cryo-EM) to solve the structures of *Drosophila melanogaster* kinesin-13 KLP10A protein constructs in complex with curved tubulin protofilaments or bound to the microtubule lattice. Our results show how the kinesin-13-specific loop-2 couple ATP-hydrolysis conformational changes in the motor domain with tubulin bending and microtubule depolymerization.

## Results and Discussion

### KLP10A in complex with straight or curved tubulin

**Fig. 1:**
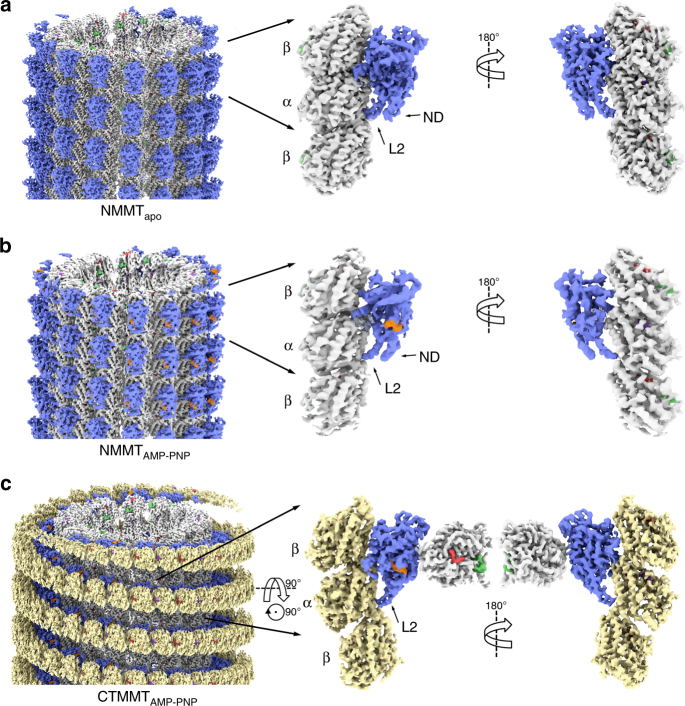
Cryo-EM 3D density maps. **a** NMMT_apo_ complex. **b** NMMT_AMP-PNP_ complex. **c** CTMMT_AMP-PNP_ complex. Left shows the whole helical 3D map, center and right shows two orientations of one asymmetric unit with an extra β-tubulin subunit. Coloring was done based on the fitted atomic models: microtubule lattice tubulin (straight tubulin) in white-gray, wrapping tubulin protofilament (curved tubulin) in ochre, KLP10A in blue, AMP-PNP in orange, GTP in purple. GDP in red, and paclitaxel in green. Note, that the interaction of the KLP10A motor-domain with straight tubulin in the NMMT complexes (**a**, **b**) and with curved tubulin in the CTMMT_AMP-PNP_ complex (**c**) are through the same putative kinesin–tubulin interface. An additional kinesin-13-specific tubulin binding site^11,19^ mediates the interaction of the motor domain with the microtubule in the CTMMT_AMP-PNP_ complex

In the presence of ATP-like analogs kinesin-13 protein constructs that include the motor domain bind to the microtubule lattice and also form stable and very ordered complexes with curved tubulin. The complexes with curved tubulin consist of curved protofilaments with bound kinesins and can be observed by electron microcopy in isolation, bound at the end of microtubules or wrapped around microtubules forming spirals^9–11,18–20^. Examples of these complexes obtained with the KLP10A protein constructs used in this work are shown in Supplementary Fig. 1. On the other hand, in the absence of nucleotides (apo-state), KLP10A does not form complexes with curved protofilaments^10^, but binds well to straight tubulin in the microtubule lattice (Supplementary Fig. 1a). We solved the cryo-EM structures of minimal microtubule-depolymerization-capable kinesin-13 protein constructs in complex with curved tubulin protofilaments or bound to the microtubule lattice (Fig. 1, Table 1). The protein constructs based on the *Drosophila melanogaster* kinesin-13 KLP10A include either the motor domain alone (M) or the motor plus the kinesin-13-specific neck-domain (NM: motor plus 81 residues N-terminus of the motor-domain). We solved the structures of three complexes covering distinct nucleotide states and tubulin conformations: a complex formed by the NM construct bound to the microtubule lattice (straight tubulin) in the absence of nucleotides (NMMT_apo_, Fig. 1a); a complex formed by the NM construct, bound to the microtubule lattice in the presence of the non-hydrolysable ATP analog AMP-PNP (NMMT_AMP-PNP_, Fig. 1b); and a complex formed by the M construct, bound to curved tubulin (CT) protofilaments wrapped around microtubules in the presence of AMP-PNP (CTMMT_AMP-PNP_, Fig. 1c). The overall resolution of the cryo-EM 3D density maps (3.5–4.0 Å overall and 3.8–4.8 Å in the kinesin parts) was sufficient to build atomic models in which the polypeptide chains were fully traced and the position of the ligands and many of the amino acid side chains determined (Fig. 1, Table 1, Supplementary Figs. 2 and 3). Comparing the kinesin and tubulin structures in the three complexes revealed conformational changes associated with nucleotide binding and how they are coupled to the bound tubulin conformation.

**Table 1:** Cryo-EM data collection, refinement, and validation statistics

	#1 NMMT_apo_ (EMDB-7027) (PDB 6B0I)	#2 NMMT_AMPPNP_ (EMDB-7028) (PDB 6B0L)	#3 CTMMT_AMPPNP_ (EMDB-7026) (PDB 6B0C)
Data collection and processing			
Nominal magnification	22,500	22,500	22,500
Voltage (kV)	300	300	300
Electron exposure (e^–^/Å^2^)	63.8	63.5	62.5
Defocus range (μm)^a^	1.3–2.2	1.1–2.3	1.1–2.5
Pixel size (Å)	1.073	1.073	1.073
Number of images taken	449	1464	2110
Number of images used	229	202	482
Symmetry imposed	Helical	Helical	Helical
Initial particle images (no.)^b^	23,986	30,971	21,134
Final particle images (no.)^c^	10,647	6040	16,942
Number of asymmetrical units	159,705	90,600	254,130
Map resolution (Å)	3.78	3.98	3.51
FSC threshold	0.143	0.143	0.143
Map resolution range (Å)^d^	2.8–550	3.0–550	2.6–660
Refinement			
Initial model used (PDB code)	See note e	See note e	3J2U
Model resolution (Å)	3.78	3.98	3.51
FSC threshold	0.143	0.143	0.143
Model resolution range (Å)	3.78	3.98	3.51
Map sharpening *B* factor (Å^2^)	−130	−20	−100
Model composition			
Non-hydrogen atoms	9829	9861	16,440
Protein residues	1238	1238	2060
Ligands	3	4	6
R.m.s. deviations			
Bond lengths (Å)	0.011	0.012	0.009
Bond angles (°)	1.38	1.33	1.22
Validation			
MolProbity score	1.83	1.97	1.72
Clashscore	6.59	8.67	5.43
Poor rotamers (%)	0.19	0	0.17
Ramachandran plot			
Flavored (%)	92.53	91.64	93.40
Allowed (%)	100	100	99.95
Disallowed (%)	0	0	0.05

^a^ Range of the average defocus measured on micrographs. The range comprises 90% of the micrographs used (5% of the micrographs defocuses are below and 5% are above this range)

^b^ Total number of particles picked (Methods)

^c^ Number of 15R particles retained after the second classification round (Methods)

^d^ Higher resolution estimate (lowest spacing value) corresponds to the lowest spacing bin containing at least 1% of the voxel boxes of the blocres local resolution distribution (see Methods and Supplementary Fig. 3)

^e^ Computationally generated models based on the KIF14 complex with a 15 protofilaments microtubule map (EMD 2609), tubulin PDB file 1JFF, and kinesin PDB file 4OZQ

### Confomational changes in the nucleotide binding pocket

**Fig. 2:**
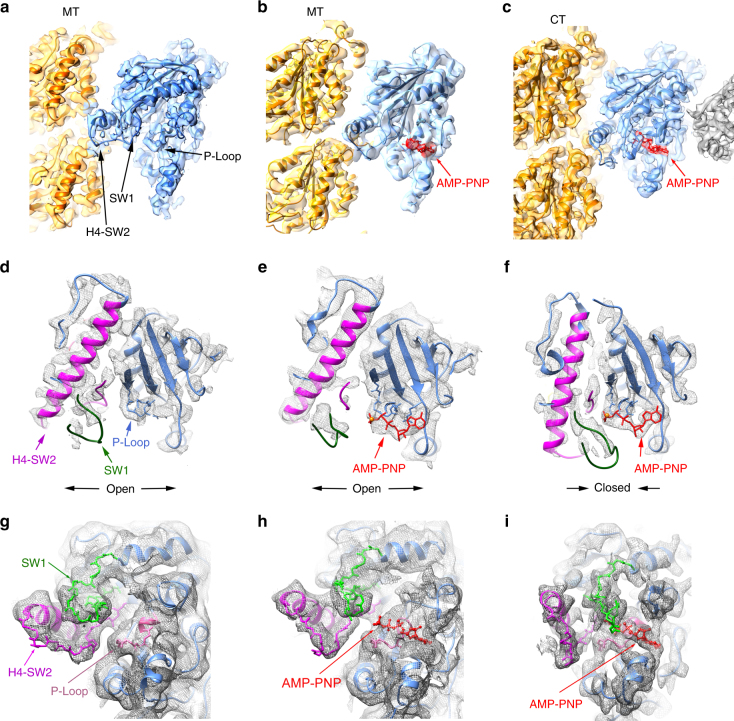
KLP10A nucleotide binding pocket. **a**–**c** Side views (tubulin plus-end up). **d**–**f** Top view detail of the KLP10A nucleotide binding pocket. **g**–**i** Side view detail of the KLP10A nucleotide binding pocket. **a, d, g** NMMT_apo_. **b, e, h** NMMT_AMP-PNP_. **c, f, i** CTMMT_AMP-PNP_. In **a**, **b**: KLP10A blue; tubulin yellow; cryo-EM density as a gray semi-transparent surface. In **c**–**i**: KLP10A blue, H4-SW2 magenta, SW1 green, P-loop pink, AMP-PNP red, cryo-EM density as a gray mesh surface

The structure of the KLP10A nucleotide binding site can be described in terms of the common structural elements found in other kinesins and the related NTPases, myosins, and G-proteins^21,22^. These elements include: the P-loop, a central β sheet with flanking α-helices, and the switch loops (SW1 and SW2). The P-loop is involved in nucleotide binding and the switch loops sense the nucleotide species present in the active site by adopting distinct conformations^21^. In all three 3D maps, the switch loops are resolved (Figs. 1 and 2). In the NMMT_apo_ complex, the nucleotide binding pocket is empty and the electron densities associated with switch loops regions are positioned relatively far away from the nucleotide site (Fig. 2a, d, g). In analogy with other NTPases this corresponds to the “open” configuration of the catalytic site. Interestingly, the catalytic site is also in the open configuration in the NMMT_AMP-PNP_ structure despite the presence of AMP-PNP in the nucleotide binding pocket (Fig. 2b, e, h). In contrast, in the CTMMT_AMP-PNP_ complex the KLP10A nucleotide binding pocket adopts the “closed” configuration (Fig. 2c, f, i). The switch loops move closer to the nucleotide γ-phosphate and α-helix 4 (H4) which is part of SW2 adopts a different orientation relative to the rest of the motor domain (Fig. 2, Supplementary Movies 1-3).

The closed configuration of the nucleotide binding pocket found in the CTMMT_AMP-PNP_ complex is similar to the one found in the crystal structures of motile kinesins thought to represent the hydrolysis competent ATP bound form^23^. It is also similar to the crystal structure reported recently of another kinesin-13 (hMCAK) in complex with curved tubulin^24^. These similarities indicate that the nucleotide pocket of kinesin-13s and motile kinesins undergo similar conformational changes in response to the nucleotide species in the active site. However, our results also indicate that in kinesin-13s these conformational changes are coupled not only to the nucleotide species in the active site but also to the particular tubulin configuration, straight or curved to which the motor domain is bound. The nucleotide pocket is closed with AMP-PNP only when bound to curved tubulin and it is open when bound to straight tubulin (Fig. 2, Supplementary Movies 1-3).

Near the nucleotide binding pocket we also observed structural differences between KLP10A and other kinesins that are likely related to the distinct functionality of kinesin-13s. Loop-5, near the P-loop, is in a different configuration than the one observed in other kinesins, where it is either not resolved or it is in a different configuration than the one observed here (Supplementary Fig. 4). Residues in this area are not highly conserved between kinesin families and it is thought that distinct L5s modulate the ATPase activity of different kinesins^25^. Accordingly, the distinct configuration observed here may be partially responsible for the unique ATPase properties of kinesin-13s^26,27^.

**Fig. 3:**
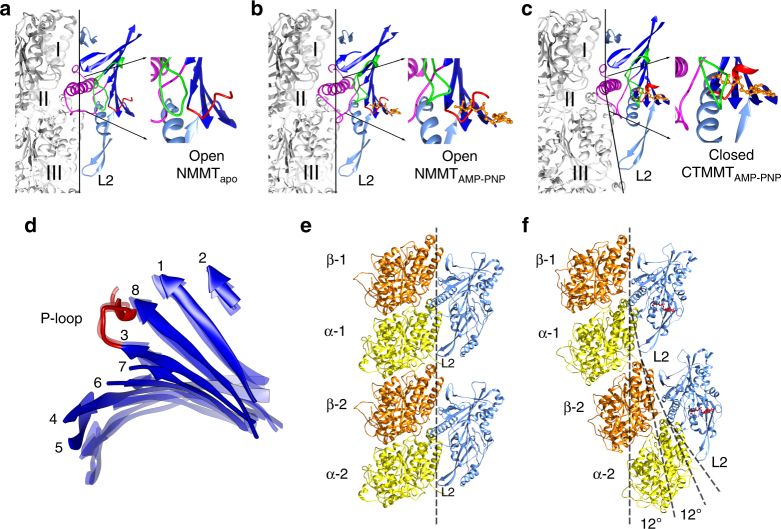
Reshaping of the KLP10A–tubulin interface. **a**–**c** Side views highlighting selected areas of the KLP10A motor domain and tubulin interface undergoing conformational changes. Inset at the right of each panel shows a zoomed view the nucleotide pocket. Central β-sheet strands in dark blue; Switch-1 loop in green, Switch-2 and H4 in magenta, L2, L7, L8 and H6 in light blue. The three areas of tubulin–KLP10A interaction (I–III) are indicated. Note the line connecting the three interacting areas highlighting their different relative positions in the NMMT_apo_ and NMMT_AMP-PNP_ (**a**, **b**) relative to the CTMMT_AMP-PNP_ complex (**c**). **d** P-loop aligned central β-sheets of the three complexes, NMMT_apo_ and NMMT_AMP-PNP_ in light semi-transparent blue and CTMMT_AMP-PNP_ in dark blue. Numbers indicate the strand order in the KLP10A sequence. **e, f** Superimposed KLP10A model structures aligned on the top β-tubulin subunit (β-1). **e** NMMT_apo_. **f** CTMMT_AMP-PNP_. Closing of the KLP10A nucleotide pocket is associated with a ~12° rotation of between monomers of the bound tubulin (24° per tubulin heterodimer)

Movement of the switch regions relative to the nucleotide and P-loop causes a rearrangement of sub-domains within the KLP10A motor domain. The central eight stranded β-sheet, which contains the P-loop and spans several regions within the motor domain, becomes less twisted in the CTMMT_AMP-PNP_ complex relative to the NMMT_apo_ and NMMT_AMP-PNP_ complexes (Fig. 3a–d). Distinct twist of the central β-sheet strands in different nucleotide conditions has also been observed in myosin^22^ and other kinesins^28^. The reorientation of H4, which is part of the kinesin–tubulin interface in the complexes, causes a reorientation of the rest of the motor domain relative to the bound tubulin. These movements between domains alter the position of loop-2 relative to other areas of the motor domain (Fig. 3a–c). The ultimate consequence of all these structural differences is a reshaping of the tubulin binding interface so that it alternatively complements the shape of straight or curved tubulin (Fig. 3e–f).

### The KLP10A–tubulin interface

**Fig. 4:**
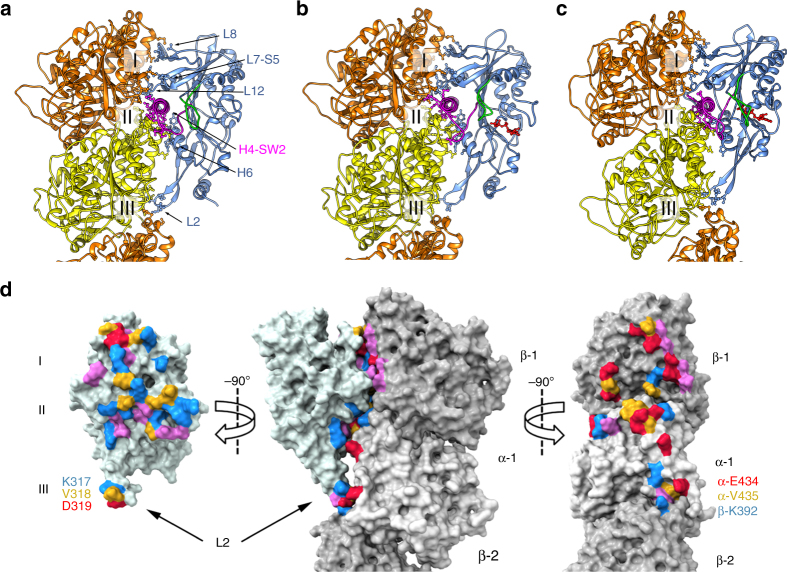
**a**–**c** KLP10A-tubulin binding interface. Side views of the complexes indicating KLP10A secondary structure elements making contact with tubulin in the three areas of interaction (I–III). **a** NMMT_apo_. **b** NMMT_AMP-PNP_. **c** CTMMT_AMP-PNP_. KLP10A in blue, SW1 in green, SW2-H4 in magenta, AMP-PNP in red, β-tubulin in orange, α-tubulin in yellow. **d** Solvent accessible surface of the CTMMT_AMP-PNP_ complex highlighting residues at the tubulin KLP10A interface within contact distance (specific residues making contacts are listed in Supplementary Fig. 5). Residues are color coded according to type. Negative red, positive blue, polar magenta, and non-polar yellow

The KLP10A motor domain putative tubulin binding interface can be subdivided in three areas (Figs. 3, 4): Area I, comprising of interactions with β-tubulin, area II comprising of interactions with α- and β-tubulin at the intra-dimer interface, and area III consisting of interactions with α- and β-tubulin at the inter-dimer interface. Area III is formed by the kinesin-13 class-conserved elongated loop-2 (L2). The interacting residues comprise the kinesin-13 class conserved motif KVD at the L2 tip and complementary residues located in α- and β-tubulin (Fig. 4d). This interaction is critical for kinesin-13 function as mutating the residues KVD to alanines abolishes kinesin-13 microtubule depolymerase activity^11,29,30^. The position of the L2 tip relative to the other two areas of interaction (I and II) is different in the CTMMT_AMP-PNP_ and the NMMT complexes (Fig. 3a–c). Despite the different L2 relative position, interactions with tubulin are maintained by a concomitant tubulin conformational change going from the straight (NMMT complexes) to the curved configuration (CTMMT_AMP-PNP_ complexes, Fig. 3, Supplementary Movies 4 and 5).

### Conformation and position of the KLP10A-neck-domain

**Fig. 5:**
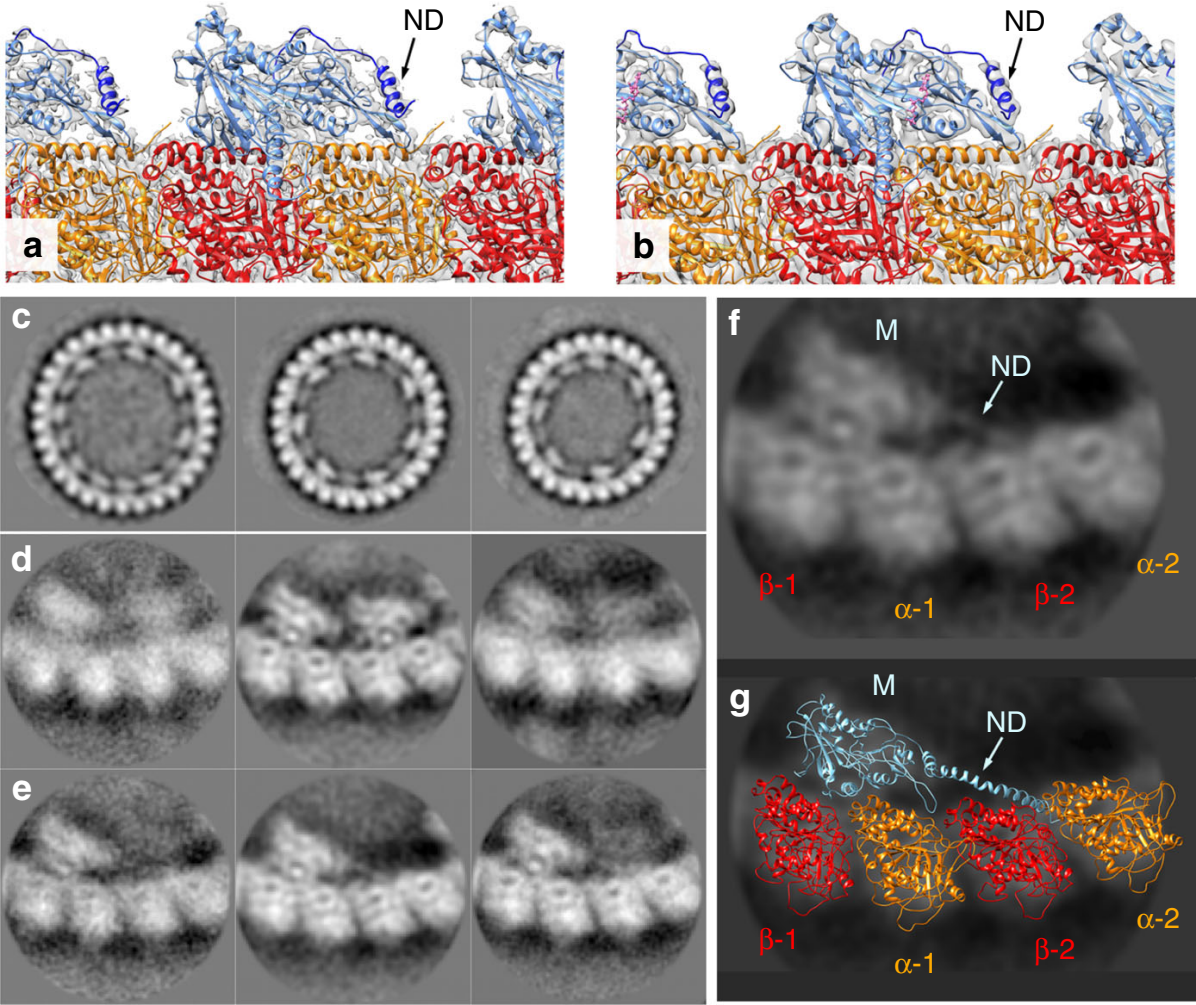
Configuration of the KLP10A neck domain. **a, b** Structure of the neck domain (ND) in the NMMT complexes. **a** NMMT_apo_. **b** NMMT_AMP-PNP_. **c** Class averages of curved tubulin protofilaments obtained by aligning and classifying whole ring-like structures observed in cryo-EM images (Supplementary Fig. 1d). **d, e** Class averages of curved tubulin protofilaments obtained by aligning/classifying regions containing two tubulin heterodimers from the classes shown in **c**. This classification produced class-averages with one KLP10A motor domain per tubulin heterodimer (**d**) and class averages with one KLP10A motor domain per two tubulin heterodimers (**e**). **f** In the class average images with only one KLP10A motor domain per two tubulin heterodimers, an elongated density can be observed going from the motor domain density (M) to the density corresponding to the next tubulin heterodimer (α-2, β2). **g** The elongated density in **f** is modeled as an α-helix formed by the KLP10A neck-domain (ND)

Thirty six residues of the neck-domain are resolved in the NMMT complexes forming a loop and an α-helix at the back of the motor domain (Fig. 5a, b). A similar structure for this part of the neck domain has been observed in the crystal structures of other kinesin-13s but with a variable orientation of the α-helix relative to the motor domain (e.g., 2GRY, 2HEH, 1V8K^29^, 5MIO^24^). To determine the position of the neck-domain in KLP10A-curved tubulin complexes we performed 2D analysis of the curved tubulin complexes not wrapped around microtubules (Fig. 5c–g). Kinesin-13 protein constructs that include the neck-domain can also form curved tubulin protofilament that wrap around microtubules^18^ but we found that the resulting complexes were usually shorter and with less helical order. This prevented their use for high resolution 3D helical reconstruction as performed here for the CTMMT_AMP-PNP_ complex. In 2D class averages of curved tubulin NM complexes two distinct decoration patterns were observed: (1) a pattern where a KLP10A motor domain binds to every tubulin heterodimer (Fig. 5d). This decoration pattern is similar to what is observed with the motor-domain only KLP10A construct^10^. (2) A skipping pattern where the motor domains bind to every other tubulin heterodimer (Fig. 5e). This pattern is similar to the one previously reported for another kinesin-13 construct that also included the neck-domain^31^. The skipping pattern occurring with kinesin-13 constructs that include the neck-domain indicates that this domain may sterically prevent the binding of two contiguous kinesin-13 molecules along a protofilament. Supporting this interpretation class average images of KLP10A NM tubulin complexes with the skipping pattern reveal an elongated density going from the back of the motor domain to the intra-dimer interface of the next tubulin heterodimer (Fig. 5e–f). This 2D density can be well fitted with a model in which the C-terminal half of the neck -domain (the part closer to the motor domain) has the same structure observed in the NMMT complexes and the N-terminal part forms an α-helix that goes from the back of the motor domain to the intra-dimer interface of the next tubulin heterodimer (Fig. 5g). This model is consistent with the predicted α-helical structure of the neck-domain sequence, but 3D data for this part of the complex would be required to fully determine its structure. The modeled configuration of the neck domain explains the skipping pattern as its position would overlap with the binding site of another motor-domain. It also supports the proposed role of the neck-domain in assisting kinesin-13 microtubule binding^31–33^. The data also indicate that the position of the neck-domain is highly variable as it can be displaced by other motor domains as indicated by the non-skipping binding pattern (Fig. 5d). We also interpret the lack of density corresponding to the N-terminal half of the neck domain in the NMMT complexes (Fig. 5a, b) as the result of this part becoming delocalized when displaced from its tubulin binding site by an adjacent motor domain bound to the microtubule lattice.

The interaction of the neck domain with an adjacent tubulin heterodimer suggests that it may play a role in inducing or increasing tubulin inter-dimer curvature. However, the neck domain is unlikely to be the major driver of tubulin curvature as the M construct can readily form stable complexes with curved tubulin.

### Tubulin conformational changes

The differences between the curved tubulin protofilament (CTMMT_AMP-PNP_ complex) and the microtubule lattice straight tubulin can be described to a first approximation as rigid body rotation of 12° between tubulin subunits (24° per tubulin heterodimer) and a small displacement between subunits perpendicular to the rotation plane. (Fig. 3e, f and Supplementary Fig. 6). We previously referred to this displacement as shear, but the amount of shear estimated here is less than previously estimated from lower resolution data^10^. The tubulin structure in the new KLP10A-curved tubulin protofilament model is relatively similar to several tubulin crystal structures but with a higher degree of curvature (Supplementary Fig. 6). The higher curvature suggests that KLP10A can bend tubulin protofilaments beyond the typical tubulin curvature observed outside the microtubule lattice. Furthermore, the 24° per dimer curvature observed in the CTMMT_AMP-PNP_ complex (helical path with 23.82° rotation and 11.1 Å rise per tubulin dimer) is not the maximum value we observe. In KLP10A–tubulin complexes not wrapped around microtubules a range of curvatures were observed up to 27° per tubulin heterodimer (Fig. 5c). The increased tubulin curvature induced by KLP10A would further disrupt the formation of lattice type tubulin lateral contacts and facilitate microtubule depolymerization.

In addition to the described rigid body movements between tubulin monomers, there are also small structural changes within the tubulin sub-units. The root-mean squared deviation (RMSD) between equivalent α-carbon positions of straight and curved tubulin were 1.9 Å and 2.0 Å for the α- and β-tubulin subunits respectively. A conformational change to highlight is the disruption of the binding site of the anti-cancer drug paclitaxel (Taxol^®^) on the β-subunit of the KLP10A bound curved tubulin. Despite all complexes being made in the presence of paclitaxel (Methods) its corresponding density is absent in the curved tubulin and the binding site is partially blocked by β-tubulin residues (Supplementary Fig. 7). In contrast, a paclitaxel associated density is present in the microtubules of all three complexes investigated (Fig. 1 and Supplementary Fig. 7). This includes the microtubule in the CTMMT_AMP-PNP_ complex that also contains the sans-paclitaxel curved tubulin.

### Comparison with other kinesin structures

To our knowledge in all the structures available of kinesin13s motor domains not bound to tubulin (e.g., PDB accession codes: 1V8K^29^, 2GRY, 2HEH, 1V8J^29^, 4UBF, 4Y05^34^, 5XJA^35^, 5XJB^35^) the switch loops appear disordered and the nucleotide binding pocket is in the open configuration, even when ATP analogs are bound. This differs from the KLP10A–tubulin complex structures presented here where the switch loops are ordered (Fig. 2g–i), whether the nucleotide binding pocket is open (NMMT complexes) or closed (CTMMT_AMP-PNP_ complex). This indicates that tubulin binding induces ordering of the switch loops. This is readily accounted for by the multiple interactions that SW1 and H4-SW2 make with each other and with tubulin in area II of the KLP10A–tubulin interface (Fig. 4 and Supplementary Fig. 5).

**Fig. 6:**
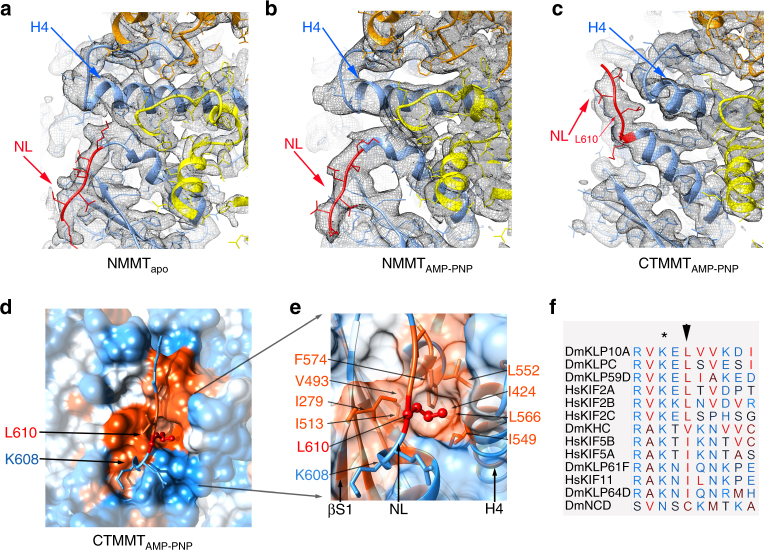
KLP10A neck-linker docking. The figure shows the structure of the beginning of the neck-linker domain present in the KLP10A protein constructs (six residues at the C-terminal end of the motor domain). **a** NMMT_apo_. **b** NMMT_AMP-PNP_. **c** CTMMT_AMP-PNP_ Neck-linker in red, KLP10A motor domain in blue, β-tubulin in orange, α-tubulin in yellow, corresponding cryo-EM map density as a gray mesh. **d** CTMMT_AMP-PNP_. structure with neck-linker in the up docked position. Core motor domain solvent exclusion surface colored by hydrophobicity with neck-linker shown as ribbon and atom sticks (Kyte and Doolittle hydrophobicity scale with orange to blue representing more to less hydrophobicity). Residue L610 colored red and shown as a ball and stick atom representation. **e** Close up of the image **d** showing the KLP10A residues forming the hydrophobic pocket where residue L610 plugs into. Residue K608 marks the end of α-helix 6 and the beginning of the neck linker. **f** Aligned neck linker sequences of six kinesins-13s (DmKLP10A, DmKLP59C, DmKLP59D, HsKIF2A, HsKIF2B, and HSKIF2C) and seven motile kinesins (DmKHC, HsKIF5B, HsKIF5A, DmKLP61F, HsKIF11, DmKLP64D, and DmNCD). Dm *Drosophila melanogaster*, Hs *Homo sapiens.* Asterisk is aligned with DmKLP10A K608. Arrow is aligned with DmKLP10A L610. Structures oriented in **a**–**e** with the tubulin plus-end at the top. *H4* α-helix 4, *NL* Neck-linker, *βS1* β-strand 1

When comparing the structure of KLP10A–tubulin complexes with that of motile kinesin such as kinesin-1 there are many similarities as expected from the high degree of homology within the motor domain in the kinesin superfamily. Areas I and II of the interface with tubulin are very similar with several kinesins conserved residues establishing contacts (Supplementary Fig. 5). The open and closed configurations of the nucleotide binding pocket and associated sub-domains movements within the motor domain^36–38^ are also very similar (Supplementary Fig. 8). In the case of N-terminal-motor-domain-kinesins, such as kinesin-1, closing of the nucleotide pocket is associated with ATP binding and docking of the neck-linker^23,36,37^, a structure C-terminal of the motor domain between the motor domain and the dimerization domain (not to be confused with the kinesin-13 neck-domain). Docking of the neck linker of one motor domain biases movement of the partner motor domain towards the microtubule plus-end driving unidirectional movement along microtubules^39^. It is also thought that the activities of the two motor domains are coordinated by the position of their connecting neck linkers^40^. In the case of C-terminal-motor-domain-minus-end-directed kinesins, docking of their C-terminal region (corresponding to the neck linker) is associated with a reorientation of the stalk domain (located N-terminus to the motor domain) in the microtubule minus-end direction^22,41^. Interestingly, we also observe docking of the neck linker associated with closure of the nucleotide binding pocket (Fig. 6). The KLP10A neck-linker is in a down orientation (pointing toward the microtubule minus end) in the structures where the nucleotide binding pocket is open (Fig. 6a, b) and docks in an up orientation when the nucleotide binding pocket is closed (Fig. 6c–e). As the nucleotide binding pocket closes the ensuing sub-domain re-arrangements provoke conformational changes at the other side of the motor domain where the neck-linker is located. H4 and β-strand-1 move apart creating a hydrophobic pocket where residue L610 at the beginning of the neck-linker plugs into. A hydrophobic residue at the equivalent position of KLP10A L610 is highly conserved among kinesins (Fig. 6f), and a similar hydrophobic interaction between the neck-linker (or C-terminal end in C-terminal kinesins) and the motor domain is observed in the structures of motile kinesins thought to represent the ATP bound form of the motor domain^22,37,41^. These results indicate that the microtubule depolymerizer kinesin-13s and the motile kinesins share the same basic neck-linker docking mechanism. In the case of kinesin-13s neck-linker docking appears unlikely to be the conformational change directly leading to microtubule depolymerization. However, as in motile kinesins it may help to lock the motor domain in the nucleotide-pocket-closed catalytic configuration. It may also provide a mechanism for coordinating the activity of the two motor domains in a full length kinesin-13 dimer^42^.

Coordination between partner motor domains through their neck-linkers connection allows kinesin-1s and other microtubule plus-end-directed motile kinesins to walk processively along microtubules^43^. Full length kinesin-13s are also reported to be moderately processive enzymes that on average remove four tubulin dimers per encounter with a microtubule end^15^. As with N-terminal kinesins such as kinesin-1, the kinesin-13s dimerization domain (or part of it) is located C-terminal to the motor domain^12,31^. This configuration makes the two neck linkers of the partner motor domains the inter-connecting structural element that provides a communication pathway between the two nucleotide binding pockets. The fact that KLP10A and kinesin-1s show a similar relationship between neck-linker position and nucleotide binding structure (open or close) raises the possibility that similar mechanisms mediate processivity in motile and microtubule depolymerase kinesins.

### Microtubule depolymerization mechanism

**Fig. 7:**
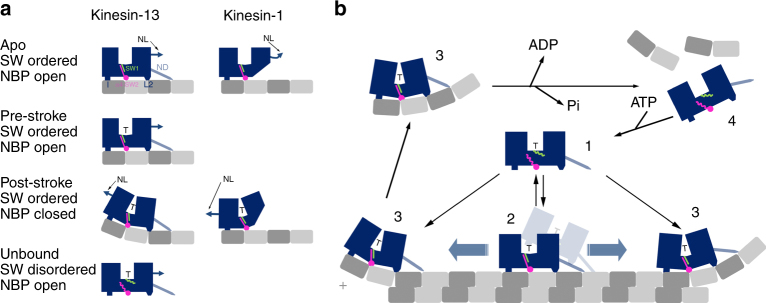
Kinesin-13 microtubule depolymerization model. **a** Cartoon representing four distinct kinesin-13 conformations and equivalent states in kinesin-1: apo as in the NMMT_apo_ complex; pre-stroke as in the NMMT_AMP-PNP_ complex; post-stroke as in the CTMMT_AMP-PNP_ complex; unbound as in the structures of several kinesin-13 motor domains not bound to tubulin. The switch regions are represented as green and magenta lines with straight or wavy lines indicating ordered or disordered conformations respectively. The three areas of interaction with tubulin are represented as a square (area I), a circle (area II, H4, SW2), and a trapezoid (area III, L2). The neck-domain is represented as a light blue line. Closing of the nucleotide pocket and reshaping of the tubulin interface is represented as a bending motion between the two halves of the motor domain (blue). *SW* switch regions, *NBP* nucleotide binding pocket, *ND* Neck-domain, *NL* Neck-linker, T: ATP. **b** Proposed model for kinesin-13 microtubule depolymerization and ATPase activity coupling (neck-linker on the KLP10A motor domain cartoon representation not shown). See main text for discussion

We propose that the key conformational change leading to tubulin bending and microtubule depolymerization is the movement of the kinesin-13-specific loop-2 relative to the other areas of the kinesin-13–tubulin interface (Fig. 7). The most significant differences between KLP10A and kinesin-1–tubulin complexes are the additional tubulin contacts made by L2 and the relationship between nucleotide species, tubulin binding and nucleotide pocket closure (Supplementary Figs. 5, 8, 9). In the case of KLP10A, nucleotide pocket closure requires ATP binding (as mimicked by AMP-PNP) and interaction with curved tubulin as observed in the CTMMT_AMP-PNP_ complex. In the NMMT_AMP-PNP_ complex, the nucleotide pocket remains open despite AMP-PNP being present in the active site and the switch loops being ordered. On the other hand in the case of kinesin-1 the nucleotide pocket closes with ATP binding while bound to the microtubule-lattice-straight tubulin^44^. Given that ATP hydrolysis is thought to be catalyzed by the closed configuration of the nucleotide pocket^23^, our structures imply that KLP10A is not catalytic when bound to the microtubule-lattice and becomes catalytic when bound to curved tubulin. This provides a structural explanation to the reported higher kinesin-13 ATPase stimulation by the microtubule ends relative to the microtubule lattice^14^.

Based on our results we propose the following model for kinesin-13 induced microtubule depolymerization (Fig. 7). In this model sub-domains within the motor domain of kinesin-13s and motile kinesins undergo similar conformational changes related to their ATP hydrolysis cycles. However, the unique interactions of the kinesin-13 loop-2 with the tubulin inter-dimer interface provide the critical link that couples the kinesin-13 ATPase activity with tubulin bending (Fig. 7a). Loop-2 works like a latch that prevents motor sub-domain motions and nucleotide binding pocket closure while the motor domain is bound to the microtubule lattice.

When not interacting with tubulin the kinesin-13 motor domain would be predominantly in an ATP bound form^14,26,27^. ATPase activity would be low because the switch loops are disordered and the nucleotide binding pocket is in the open configurations (Fig. 7b, state 1). Binding to the microtubule lattice cause ordering of the switch regions but the nucleotide pocket is prevented from closing as the interaction of loop-2 with the tubulin inter-dimer interface prevents sub-domain movements (Fig. 7b, state 2). This results in a relatively weak interaction^27^ where the kinesin motor domain can detach from the microtubule or remain attached in a non-stereo-specific highly mobile mode that can diffuse along the microtubule lattice^33^ (one-dimensional diffusion). In this state our structural results predict that kinesin-13 stays predominantly in an ATP bound form as the nucleotide pocket is in the open configuration (non-catalytic) when bound to the microtubule lattice (Fig. 2). Alternatively, interaction with the microtubule lattice could allow closure of the binding pocket (e.g., during microtubule unbinding) leading to ATP-hydrolysis and to a diffusive ADP-Pi bound form proposed to exists in other models^15,26^. When an ATP containing kinesin-13 reaches the microtubule end, directly or by one-dimension diffusion over the microtubule lattice, it would be able to bend tubulin (Fig. 7, state 3). Kinesin-13s would be able to bend protofilaments specifically at the microtubule ends and not in the lattice, because at the ends it is expected that at least one or more protofilament would lack of a full set of lateral contacts, which presumably would make them more prone to bending. Kinesin-13s could also bind to already curved protofilaments at the microtubule ends. In either case the interaction with curved tubulin would be accompanied with closure of the kinesin-13 nucleotide pocket and catalysis activation (state 3). The kinesin-13 bound curved protofilaments could not get reincorporated into the microtubule lattice and between ATP hydrolysis and product release, they would detach from the microtubule. After product release, the motor domain would alter its shape to the apo conformation that it is no longer complementary to the curved tubulin structure. This would lead to detachment of the kinesin-13 motor domain from curved tubulin (state 4). We propose that this occur after product release as curved-tubulin–KLP10A complexes can form in nucleotide conditions mimicking the ATP, ADP-Pi, and ADP states but not the apo state^10^. Binding of ATP to the dissociated motor domain completes the cycle (State-1).

In summary, our structural studies provide an atomic level detail of the mechanism of microtubule depolymerization by kinesin-13s, how this process is coupled to ATP hydrolysis and how it relates to the mechanism of translocation by motile kinesins.

## Methods

### Kinesin proteins expression

Kinesin proteins were expressed in BL21 cells (BL21(DE3)pLysS, Thermofisher). The motor construct (M) includes *Drosophila melanogaster* KLP10A residues 279–615 and was expressed as previously reported^18^. The neck motor construct (NM) includes KLP10A residues 198–615 cloned into pRSET B plasmid with an N-terminal histidine tag. A TEV protease site (ENLYFQG) was introduced between the His tag and the start of the KLP10A sequence. Plasmid transformed cells were grown overnight in LB media containing 200 μg/ml ampicillin and 34 μg/ml chloramphenicol. The overnight culture was used to inoculate 6–8 l of LB media containing 200 µg/ml ampicillin and 34 μg/ml chloramphenicol with an initial OD_600 nm_ of 0.2–0.3. The cultures were incubated at 240 rpm, 37 °C until the OD_600 nm_ reached 1.2. Protein expression was induced by adding 700 μM IPTG and incubating for 7 h at 30 °C and 200 rpm. Cells were spun for 15 min at 3000 × *g* and the pellet stored at −80 °C. All the purification steps below were performed at 4 °C. Bacteria cells were lysed at 4 °C with a microfluidizer in buffer A (1 mM MgCl_2_, 1 mM EGTA, 250 mM KCl, 1 mM Mg-ATP, 5 mM β-mercaptoethanol, 80 mM K-PIPES, pH 7.2, adjusted with KOH) supplemented with 10 mM imidazole and one tablet of protease inhibitor cocktail (Sigmafast Protease Inhibitor Cocktail Tablets, EDTA-Free, Sigma-Aldrich) per 20 g of cell paste. The lysate was spun for 40 min at 150,000 × *g* and 4 °C. The supernatant was passed through a NiNTA column (Qiagen) that was previously equilibrated with buffer A supplemented with 10 mM imidazole. The column was washed sequentially with: first, 100 ml of buffer A supplemented with 50 mM imidazole; second, 100 ml of buffer A supplemented with 1 M KCl; third, 8 ml buffer A. Elution was performed by adding and collecting 1 ml at a time of buffer A supplemented with 300 mM imidazole. Fractions containing the proteins were pooled and subjected to dialysis overnight in buffer A with histidine-tagged TEV protease^45^ to cleave the tag (TEV/kinesin molar ratio of 1/20). TEV protease was expressed in transformed BL21(DE3)-RIL cells with plasmid pRK793^46^ (Addgene plasmid # 8827, courtesy of David Waugh lab).

After dialysis, the protein solution was passed 1 ml at a time on a 5 ml NiNTA column pre-equilibrated with buffer A supplemented with 20 mM imidazole and the eluted fraction were collected. The untagged neck-motors in the column were released by washing the column—1 ml at a time—with a total volume of 20 ml of the equilibration buffer supplemented with 20 mM imidazole. The collected fraction containing the untagged neck-motor construct were pooled, concentrated to 600 μl and further purified by size-exclusion chromatography using a Superdex 200 Increase 10/300 GL column pre-equilibrated with buffer B (1 mM MgCl_2_, 1 mM EGTA, 250 mM KCl, 150 µM Mg-ATP, 5 mM β-mercaptoethanol, 80 mM K-PIPES, pH 7.2). The purified kinesin solution was concentrated, supplemented with 20 % (v/v) sucrose, aliquoted and flash frozen at −80 °C with a final protein concentration of 140–150 μM. KLP10A concentration was estimated by UV absorbance at *λ* = 280 nm using a Nanodrop (ThermoFisher, MA) and an extinction coefficient of 23,300 l/mol/cm (value estimated from the protein sequence and assuming one ATP molecule bound).

### Microtubules

Microtubules were prepared from porcine brain tubulin (Cytoskeleton, Inc. CO). Tubulin lyophilized pellets were resuspended in BRB80 (80 mM K-PIPES, 1 mM MgCl_2_, 1 mM EGTA, pH 6.8) to 45 μM and spun at 313,000 × *g* before polymerization to eliminate aggregates. Microtubule polymerization was done in conditions to enrich the number of microtubules with 15 protofilaments^47^ as follows. The clarified resuspended tubulin solution was supplemented with GTP, MgCl_2_, DMSO to final concentrations of ~35 µM tubulin, 80 mM PIPES, pH 6.8, 1 mM EGTA, 4 mM MgCl_2_, 2 mM GTP, 12% (v/v) DMSO and incubated 40 min at 35 °C. An aliquot of stock Paclitaxel (Taxol®) solution (2 mM in DMSO) was added for a final paclitaxel concentration of 250 µM and incubated for another 40 min at 35 °C. The microtubules were then spun at 15,500 × *g*, 25 °C and the pellet resuspended in BRB80 with 20 μM paclitaxel. A slightly different microtubule polymerization solution (80 mM PIPES, pH 6.8, 1 mM EGTA, 1 mM MgCl_2_, 2 mM GTP, 20 μM paclitaxel) was used to prepare NM-curved tubulin-protofilament complexes not wrapped around microtubules (CTNM_AMP-PNP_ complex, Fig. 5). Tubulin concentration was estimated by UV spectroscopy using an extinction coefficient of 115,000 l/mol/cm on a solution with guanidine hydrochloride at a final concentration of 5.25 M.

### Cryo-EM NMMT complexes preparation

NM aliquots were thawed on ice and buffer exchanged at 4 °C to BRB40 (40 mM K-PIPES, pH 6.8, 1 mM MgCl_2_, 1 mM EGTA) supplemented with 50 μM ATP, using a spin-column (Micro Bio-Spin 6 Column, Bio-Rad) and kept on ice.

A solution containing the KLP10A NM protein construct and the appropriate nucleotide conditions to be tested (NM mix) was prepared: 88 μM NM KLP10A in BRB40 supplemented with 20 μM paclitaxel and either 20 × 10^−3^ unit of potato apyrase (Sigma-Aldrich, MO) for the apo state or 4 mM AMP-PNP (Sigma-Aldrich, MO) and 4 mM MgCl_2_ for the AMP-PNP state (all final concentrations). Four µl of a microtubule solution (8 μM tubulin in BRB80 plus 20 μM paclitaxel) were layered onto untreated EM grids (Quantifoil carbon/copper grids 300 mesh R2/2), incubated 1 min at room temperature and then the excess liquid removed from the grid using a Whatman #1 paper. Four µl of the NM mix were then applied onto the EM grid and incubated for 1 min at room temperature and then the excess liquid blotted as before. A second application of 4 µl of a freshly prepared NM mix to the grid was performed and then the grid was mounted into a Vitrobot apparatus (FEI-ThermoFisher MA), incubated 1 min at room temperature and plunge frozen into liquid ethane. Vitrobot settings: 100% humidity, 3 s blotting with Whatman #1 paper and −2 mm offset. Grids were stored in liquid nitrogen until imaging in a cryo-electron microscope.

### Cryo-EM CTMMT_AMP-PNP_ complex preparation

Microtubules and the KLP10A M protein construct were mixed in an incubation solution: 3 μM KLP10A M, 1.5 μM polymerized tubulin in BRB80, with 2 mM AMP-PNP, 2 mM MgCl_2_ and 20 μM paclitaxel (all final concentrations). The mix was incubated at room temperature for 20 min and spun for 10 min at 28 °C at 30,000 × *g*. The pellet was resuspended in one fifth of the initial volume. Four µl of the resuspended solution were layered onto an untreated electron microscope grid (Quantifoil carbon/copper grids 400 mesh R2/4) and incubated 1 min at room temperature. The grid was then placed into a Vitrobot apparatus for plunge freezing into liquid ethane. Vitrobot settings: 100% humidity, 2 s blotting with Whatman #1 paper and −2 mm offset.

### Cryo-EM CTNM_AMP-PNP_ complex preparation

NM aliquots were thawed on ice and their storage buffer was exchanged at 4 °C to BRB80 supplemented with 50 μM ATP using a spin-column (Micro Bio-Spin 6 Column, Bio-Rad) and stored on ice. Microtubules and the KLP10A NM protein were combined in an incubation mix: 15 μM KLP10A NM protein and 3 μM polymerized tubulin in BRB80 buffer supplemented with 5 mM AMPPNP, 5 mM MgCl_2_, and 20 μM paclitaxel. The mix was incubated 5 min at room temperature and then layered onto untreated electron microscope grids and plunge frozen as explained above for the NMMT complexes.

### Cryo-EM data acquisition

Data were collected at 300 kV on a Titan Krios microscope equipped with a K2 summit detector. Acquisition was controlled using Leginon^48^ with the image-shift protocol. Data collection was performed manually. For each hole selected, a picture at low magnification (2692×) was used to target one to three filaments on which image-movies were taken. Image-movies consisted of 50–60 frames of 200 ms/frame and 1.24–1.28 e^−^/Å exposure per frame. For the microtubule decorated complexes image-movies were collected within a nominal defocus range of −0.5 to −1.5 µm. This resulted in a distribution of image defocus of −1.6 ± 0.8 µm (median ± SD) as estimated with Gctf^49^. Individual NM-curved tubulin complexes were imaged at a higher defocus (−3.0 ± 0.5 µm).

### Helical-single-particle 3D reconstruction

Movie frames were aligned with motioncor2^50^ (v01-30-2017) using default parameters, generating both dose-weighted and non-dose weighted sums. CTF parameters per micrographs were estimated with Gctf^49^ (v0.50) on aligned and non-dose-weighted movies average.

Number of images, particle-boxes and asymmetric units included in the 3D reconstructions is as listed in Table 1.

A helical-single-particle approach was used to obtain 3D electron density maps of microtubules decorated with KLP10A neck-motor construct (NMMT complexes) and with KLP10A motor construct-curved tubulin protofilament spirals (CTMMT_AMP-PNP_ complex). The approach consisted of the following steps:

Step 1. Microtubules were manually picked on the aligned micrograph averages using a custom script written in the R programming language.

Step 2. Microtubule symmetry identification and selection. Atomic models of microtubules ranging from 11 to 16 protofilaments of type R or L (right or left handed protofilament twist) were generated, and converted to densities with EMAN1^51^ pdb2mrc with a voxel step size of 4 Å/voxel. The densities were low passed filtered to 15 Å and the Spider^52^ operation PJ 3Q was used to generate 200 reference projections per microtubule type. Experimental microtubule image segments were extracted and resampled to 4 Å/pixel. Segments corresponding to each experimental microtubule image were subjected to multi-reference alignment and classification with the set of model microtubule image projections (Spider^52^ AP SHC operation) to identify the best matching pairs. A microtubule type was assigned to each experimental filament image as the microtubule type with more matches to the experimental filament image segments. Only filaments with a majority of the corresponding segments identified as 15R (15 protofilaments with right handed supertwist) were selected for further processing. This procedure was repeated using as reference a 15R microtubule reconstruction generated from the experimental data as explained in step 3. At the end only microtubule segments with at least 10 successive particles identified as 15R were retained for further processing.

Step 3. Initial model. To get an initial model a first round of 3D reconstruction was performed following the procedure described in step 5 but using a single reference map instead of two independent ones. The reference maps were generated by converting to electron densities the PDB model 3J2U for the CTMMT_AMP-PNP_ complex or a modified version with kinesin motor domains docked on the microtubule for the NMMT complexes (Table 1, note e). The PDBs atomic coordinates were converted to electron densities using EMAN1^51^ pdb2mrc and low-pass filtered to 20 Å. After this first 3D reconstruction the helical symmetry parameters of the resulting maps were determined using the Relion 2.0 helix toolbox^53^. The estimated parameters, rotation (*ϕ*) and rise (*r*) per subunit were *ϕ* *=* 168.083°, *r* *=* 5.50 Å for the NMMT complexes and *ϕ* *=* 168.089, *r* *=* 5.57 Å for the CTMMT_AMP-PNP_ complex.

Step 4. Microtubule axis refinement. To limit the allowed translations of the particles in the 3D refinement reconstruction procedure (step 5) we used an iterative procedure to refine the coordinates of the microtubule axis. First the manually picked microtubules coordinates were fitted by a spline curve and interpolated to about 15 times the helical rise (82 Å). These coordinates were used to extract 300 × 300 pixels 2× binned, boxed particle images (low-pass filtered to 15 Å) which were subjected to 4 centering cycles. In each cycle the particles were aligned using the Spider (operation AP SHC) against projections of the initial model created in step 3. From the translation values obtained from the projection matching procedure a new set of axis coordinates was determined. This set was fitted to a spline curve with a maximum degree of freedom of 2, and re-interpolated every 82 nm along the axis to generate a new set of coordinates for the next cycle.

Step 5. Position-orientation refinement and 3D reconstruction. Refinement was done completely independently in two halves of the data sets using two different starting models. The two independent initial models were created from the initial model obtained in step 3 by randomizing their phases beyond 20 Å resolution using EMAN2^54^ e2proc3d.py. The data were split in half by dividing each microtubule filament in two halves. For each of the two independent refinements the data was further divided in two halves. These two quarter data sets were used to get an estimate of the resolution to which the new reference was filtered for the next refinement cycle. Refinement was done in two stages. The first using Spider^52^ and the second using Frealign^55^. An exhaustive search of the Euler angles and displacements corresponding to each particle-image was performed by multi-reference alignment against projection images of the reference model (Spider operation AP SHC) on 2× binned, CTF corrected particle images (phase flipping only). The orientation and position parameters determined were used to generate a 3D density map by back-projection of the particle-image data set. Helical symmetry was imposed using himpose^56^. This map was used as the reference model for a new round of multi-reference alignment. Three rounds of the Spider alignment procedure were performed. The resolution was estimated from the FSC curve calculated using the two 3D maps obtained from the two quarters data sets. This resulted in two independently refined 3D maps each with a resolution estimate. After three rounds of alignment the estimated resolution was 6–7 Å. The final cumulative displacement and angular rotations determined in the two Spider refinements were given to Frealign^55^ to calculate the two reference models used to perform two independent position/angular refinements and 3D reconstructions. Particle-images were extracted every 82 nm along the filaments (box sizes: 512 × 512 pixels for the NMMT datasets and 616 × 616 pixels for the CTMMT_AMP-PNP_ data set). To limit the number of overlapping particle-images in each of the two halves datasets used in each of the two independent Frealign refinements the image/particle stack were reordered (and corresponding position angular information) so that odd and even images corresponded to distinct filament quarters. For CTF correction local CTF parameters were estimated with Gctf^49^ (v0.50). Several cycles of Frealign refinement (typically 5) were run limiting the data used to 1/10 Å^−1^, until no further improvement in resolution was detected. Then additional cycles, limiting the refinement data to 1/8 Å^−1^, were run until no further improvement in resolution was detected. At the end of the last cycle, the two unfiltered half maps from each independent refinement were averaged to obtain two independently refined maps.

Step 6. Final resolution estimates. Final resolution was estimated using the FSC_0.143_ criteria (Supplementary Fig. 2) from the two independently refined maps (gold standard) using masks generated with Relion^57^ 2.0 post-process. To make these masks the maps were low-pass filtered to 12 Å, thresholded, the resulting boundaries extended by 2 pixels in all direction and smoothed with a raised cosine edge 5 pixels wide. FSC curves for distinct parts of the maps (microtubule, kinesins or curved tubulin protofilament) were generated by masking the corresponding regions of the map with local masks. These local masks were created by converting to densities the corresponding region atomic models, (EMAN1^51^ pdb2mrc) low-pass filtering and thresholding them as indicated above. The same mask was used for the two NMMT maps. Local resolution was also estimated from the two raw half maps using the Bsoft^58^ (v. 1.9.5) function blocres with a kernel size of 10 pixel (Supplementary Fig. 3).

Step 7. Map post-processing. The two independently refined raw half maps were averaged, corrected for the modulation transfer function of the K2 summit detector and sharpened to restore high resolution contrast with Relion^57^ 2.0 post-processing program. The B-factors used were: −130, −100, and −20 Å^2^ respectively for the NMMT_apo_, CTMMT_AMP-PNP_, and NMMT_AMP-PNP_ maps. The maps were then locally filtered using Bsoft blocfilt according to the local resolution estimates given by Bsoft blocres (step 6). Illustration of the 3D density maps and molecular models were prepared using UCSF-Chimera^59^ and Blender.

### 2D class averages of CTNM_AMP-PNP_ complex

Images of ring-like particles of KLP10A NM construct in complex with curved tubulin protofilaments in the presence of AMP-PNP (Supplementary Fig. 1d) were aligned and classified using Relion^57^ (v 2.0). CTF was estimated using CTFFIND4^60^. First 10,016 ring-like structures were picked manually from 464 cryo-EM images by marking their centers and masking them with a circular mask of 540 Å. This set of particles was subjected to three rounds of alignment classification and averaging with 100 class averages in each round. Only particles assigned to classes showing distinct tubulin rings with resolved tubulin dimers were kept for the next cycle. In each cycle the coordinates of the particles in the original images were recalculated based on the position of the average-image particle. This was done by applying the inverse transformation used to align the particles and any additional displacement necessary to re-center the particle in the class-average-images. The updated coordinates were used to generate a new set of particles for the next round of alignment classification and averaging. This procedure resulted in three class averages with well centered ring like structures with 15, 14, and 13 tubulin dimers (Fig. 5c). The number of particles in each of these classes were respectively 469, 2171, and 920. We then calculated the coordinates in the original images corresponding to each of two contiguous tubulin dimers in these three class averages. With these coordinate three new sets of particle images (one per each of the three ring-like class average, Fig. 5c) were generated. Each particle-image was masked with a circular mask to included only two tubulin heterodimers (176 Å mask diameter). These three data sets were subjected to three rounds of alignment classification and averaging done as before (centering the particles on the inter-dimer interface of the two tubulin heterodimers approximately at the location where the tip of KLP10A loop-2 would contact tubulin). This procedure resulted in three independently aligned groups of class average images. Two distinct types of class average images could be recognized in each of the three groups. One in which there is a kinesin density associated with each tubulin heterodimer (two kinesins and two tubulin heterodimers) and another with one kinesin per two tubulin heterodimers (Fig. 5d, e). The number of particles associated to each of these two class-average images for each of the three groups (15, 14, and 13 tubulin heterodimers ring like structures) were respectively (954, 1441), (610, 567), and (1109, 530) with the first number in parenthesis corresponding to the class with two motor-domains bound and the second number corresponding to the class with one motor domain bound.

### Model building

Atomic models of the cryo-EM density maps were built as follow. First, atomic models for each of the proteins chains involved in the complex were generated from their amino-acid sequence by homology modeling using Modeller^61^. Second, models of the complexes were built by manually placing and fitting the polypeptide chain models into the cryo-EM electron density maps using UCSF-Chimera^59^. At this step N- or C-terminal residues of the chain models falling outside the electron densities (i.e., with no associated density) were deleted from the models. Third, the complex models were flexible fitted into the electron density maps using Rosetta for cryo-EM^62,63^. For this step we first generated models using the relax-protocols and picked the models with the best scores (best matching to the cryo-EM density among the lowest 20% energy models). From these models we then generated over 100 models using the iterative-local-rebuilding protocols and picked the ones with the best scores as before. Fourth, we refined the best scored Rosetta models against the cryo-EM density maps using Phenix^64^ real space refinement tools. Fifth, the Phenix refined models were edited in Coot^65^ and UCSF-Chimera^59^ to resolve a few atomic clashes, geometry problems and areas of the model outside the electron densities. The edited models were then refined again using Phenix^64^ (step 4). Several iterations of steps 4 and 5 were performed to reach the final atomic models.

### Data availability

Data supporting the findings of this manuscript are available from the corresponding author upon reasonable request. Atomic coordinates have been deposited in the Protein Data Bank (PDB) with accession numbers 6B0C, 6B0I, and 6B0L. The corresponding cryo-EM density maps have been deposited in the Electron Microscopy Data Bank under the accession numbers EMD-7026, EMD-7027, and EMD-7028.

## Electronic supplementary material

Supplementary Information

Peer Review Report

Description of Additional Supplementary Info

Supplementary movie 1

Supplementary movie 2

Supplementary movie 3

Supplementary movie 4

Supplementary movie 5
