# Author Correction: Cryo-EM reveals the structural basis of microtubule depolymerization by kinesin-13s

**DOI:** 10.1038/s41467-018-04858-6

**Published:** 2018-07-11

**Authors:** Matthieu P. M. H. Benoit, Ana B. Asenjo, Hernando Sosa

**Affiliations:** Department of Physiology and Biophysics, Albert Einstein College of Medicine, Bronx, NY 10461 USA

Correction to: *Nature Communications* 10.1038/s41467-018-04044-8, published online 25 April 2018

The previously published version of this Article contained an error in Fig. 5. In panels f and g, the α and β symbols were swapped. The error has been corrected in both the PDF and HTML versions of the Article.

