# Supplementary material for: Cryo-EM reveals the structural basis of microtubule depolymerization by kinesin-13s
Source: Nat Commun. 2018 Apr 25;9:1662. doi: 10.1038/s41467-018-04044-8 (PMC5916938; doi:10.1038/s41467-018-04044-8)
Supplement: Supplementary file 1 — Supplementary Information [file 41467_2018_4044_MOESM1_ESM.pdf]

Cryo-EM reveals the structural basis of microtubule depolymerization by Kinesin-13s

Matthieu, P.M.H. Benoit[1], Ana B. Asenjo[1] and Hernando Sosa[1]

[1] Department of Physiology and Biophysics, Albert Einstein College of Medicine, Bronx, New York

Supplementary Figures

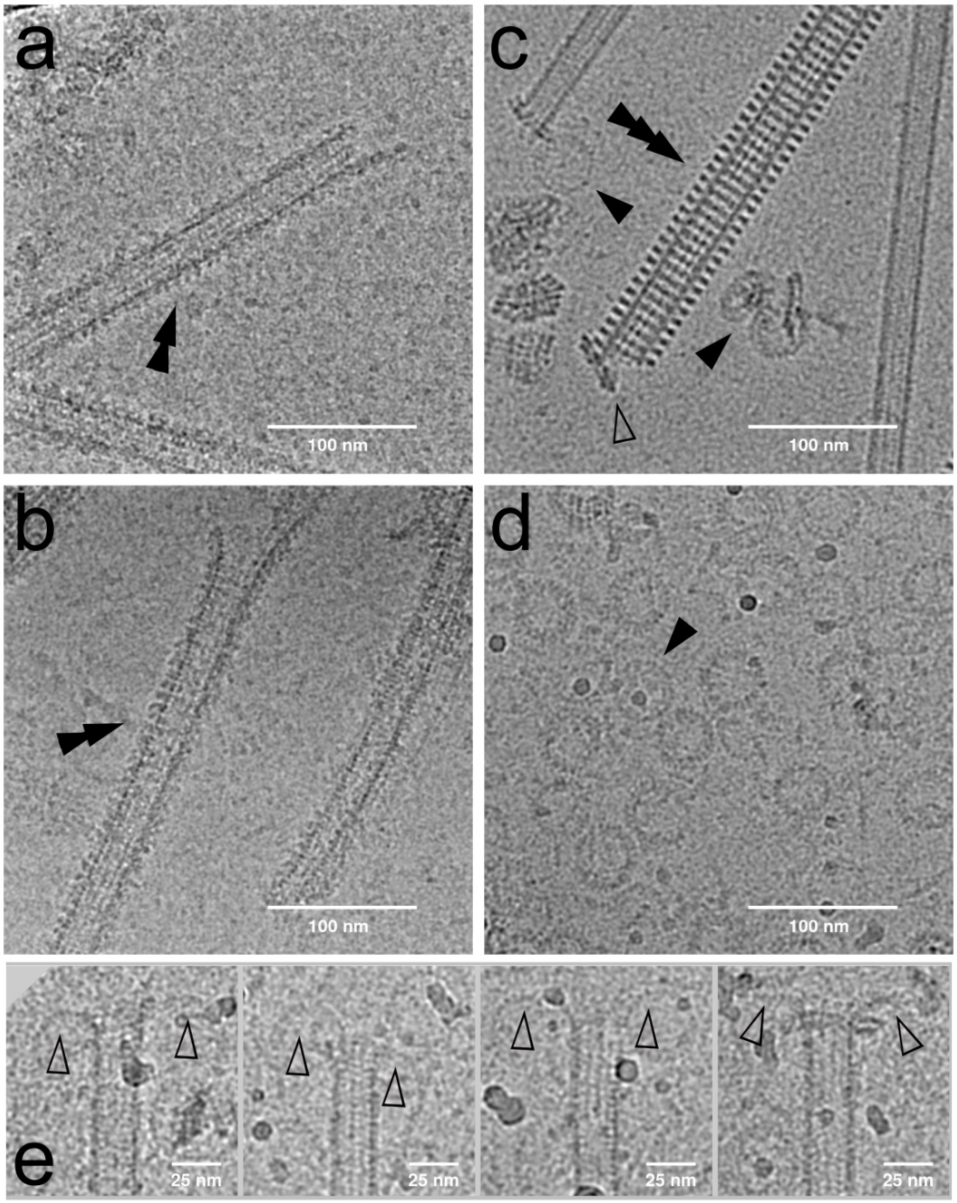

Supplementary Fig. 1. Cryo-electron micrographs. Examples of cryo-EM images (average of motion corrected movie frames). Each image corresponds to the experimental conditions used to obtain particular datasets: **a** $NMMT_{apo}$ complex. **b** $NMMT_{AMP\text{-}PNP}$ complex. **c** $CTMMT_{AMP\text{-}PNP}$ complex. **d** $CTNM_{AMP\text{-}PNP}$ complex. **e** Curved protofilaments at microtubule ends induced by the NM KLP10A construct in the presence of AMP-PNP. Double arrows in (a) and (b) show examples of microtubules with KLP10A decorated lattice. Single arrows in (c) and (d) show examples of curved-tubulin-protofilaments-KLP10A complexes. The triple arrow in (c) shows a microtubule wrapped around with curved-tubulin-KLP10A complex spirals. The open arrow in (c) and (e) shows curved protofilaments peeling away from the microtubule.

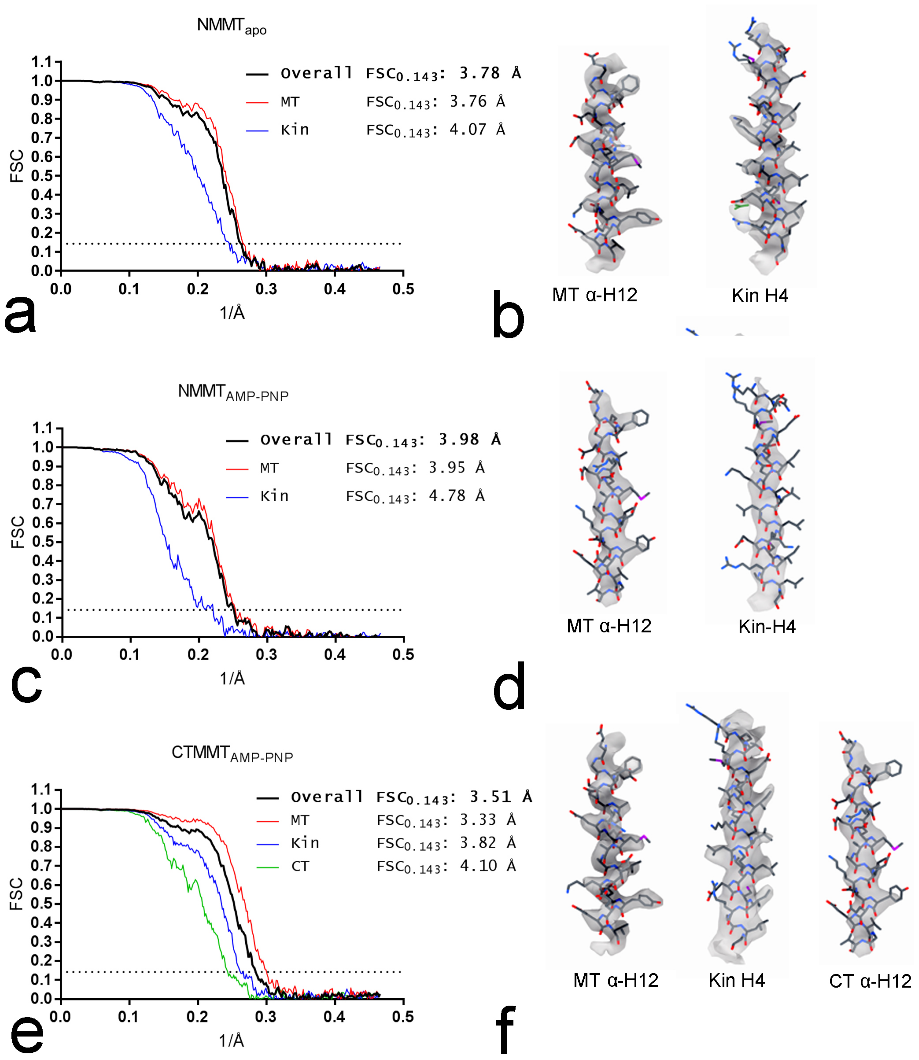

Supplementary Fig. 2 Resolution estimates. Gold standard Fourier shell correlation (FSC) of the 3D reconstructions (left) and examples of the electron densities and corresponding atomic models of some α-helices in the complexes (right). **a-b** $NMMT_{apo}$. **c-d** $NMMT_{AMP\text{-}PNP}$. **e-f** $CTMMT_{AMP\text{-}PNP}$. FSC curves corresponding to the whole complex (overall) or distinct parts of the map (MT: microtubule, Kin: KLP10A, CT: curved tubulin) are shown in different colors as indicated. The $FSC_{0.143}$ level is indicated by the dotted line.

-

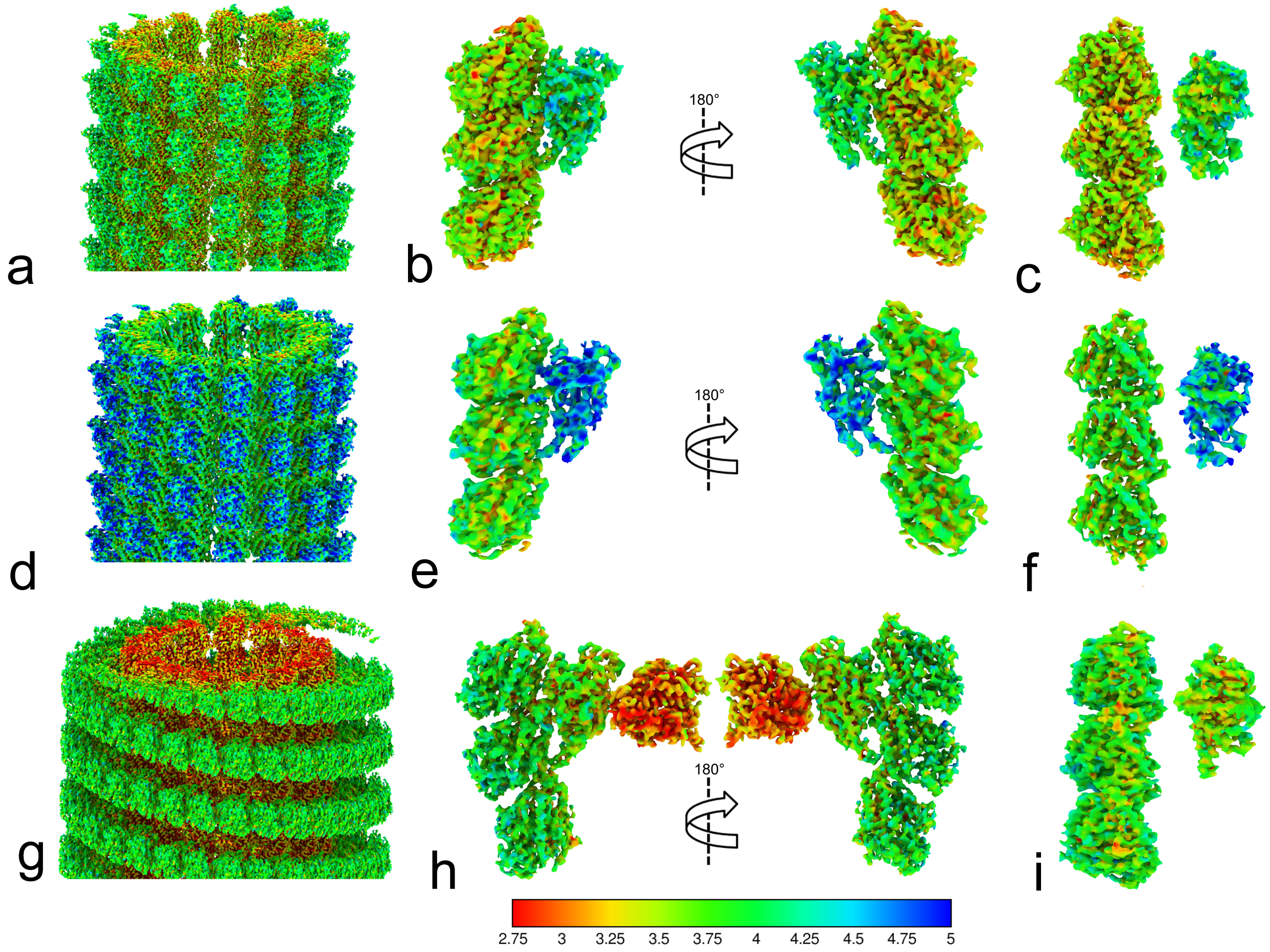

Supplementary Fig. 3 Local resolution maps. a-c NMMT$_{apo}$. **d-f** NMMT$_{AMP\text{-}PNP}$. **g-i** CTMMT$_{AMP\text{-}PNP}$. Each rows shows: the whole map (left, (a), (d) and (g)); an asymmetric unit with an extra β-tubulin subunit in two orientations (middle, (b), (e) and (h)); an asymmetric unit with an extra β-tubulin subunit showing the interface between KLP10A and tubulin (right, (c), (f), and (i)). Local resolution was estimated using Bsoft (see Methods). Resolution color scale values in Å.

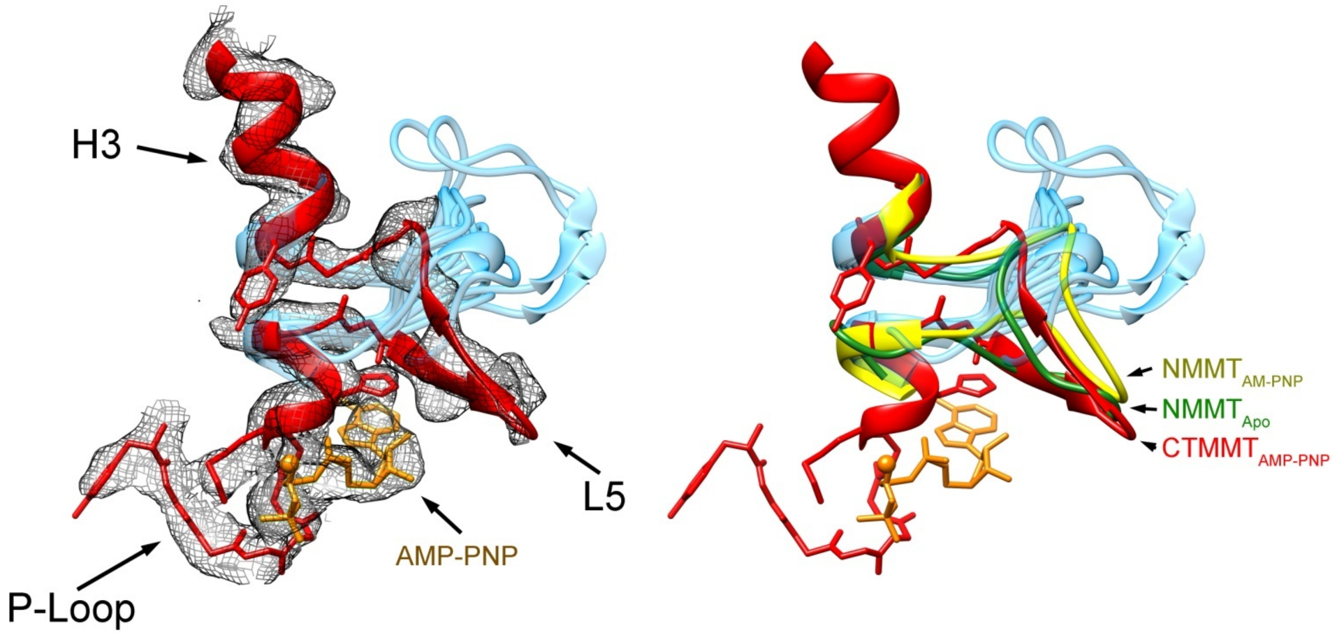

Supplementary Fig. 4 Structure of the KLP10A loop-5. The mesh shows the electron density in the helix-3 (H3) loop-5 (L5) region of the CTMMT$_{AMP-PNP}$ complex with the corresponding atomic model in red and the AMP-PNP molecule in orange. The structure of the L5 of other kinesins motor domains, after aligning the corresponding H3 and P-loop regions, are shown in light blue (PDB accession codes: 1BG2, 4HNA , 4LNU, 1MKJ , 5LT0, 5LT1, 5LT2, 5LT3, 5LT4, 3J8X, 3J8Y, 1VFW, 4OZQ, 3ZFD, 3HQD , 1Q0B, 5X3E, 3LRE , 1RY6, 2NCD). The position and orientation of L5 in the NMMT complexes are relatively similar to the one of the CTMMT$_{AMP-PNP}$ complex (right panel).

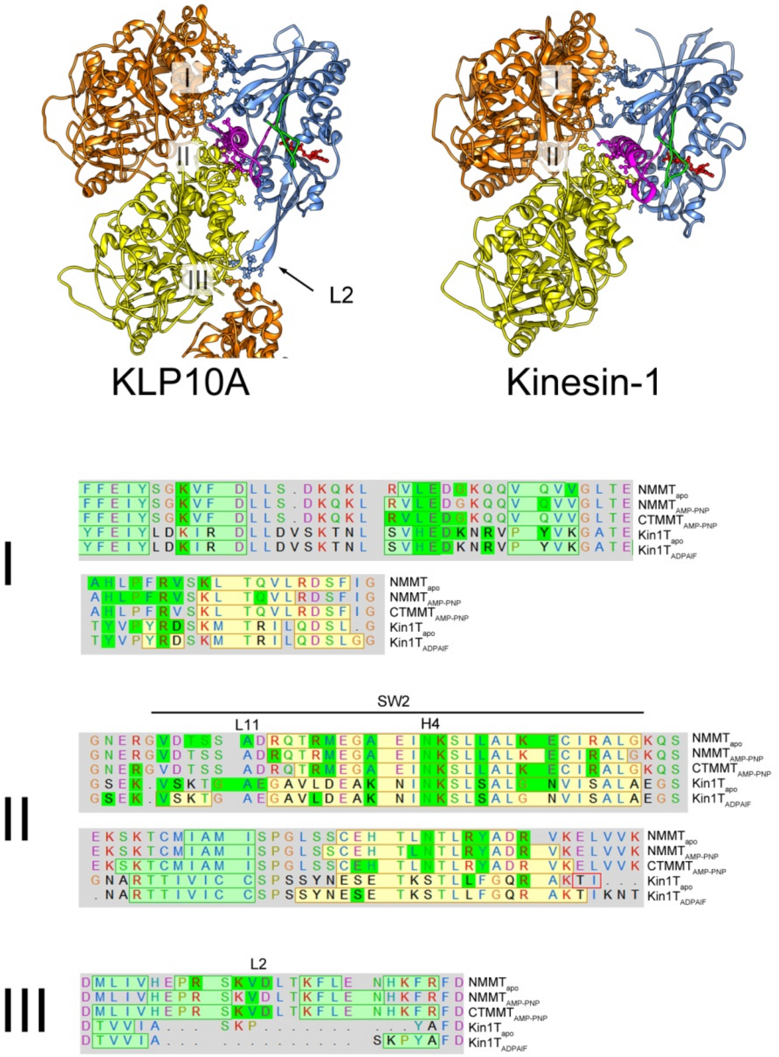

Supplementary Fig. 5 Kinesin-tubulin interactions. The top left panel: side view of the KLP10A $CTMMT_{AMP\text{-}PNP}$ complex (this work). Top right panel: side view of a kinesin-1 tubulin complex (4HNA). Kinesin-Tubulin interacting areas are divided in three regions (I, II III). Note the similarity between areas I and II between KLP10A and kinesin-1. On the other hand Kinesin-1 lacks the kinesin-13 specific long looop-2 (L2) and therefore does not interact with the tubulin inter-dimer interface (area III). Bottom panel: sequence alignment in the three kinesin-tubulin interacting regions. Kinesin residues with atoms close enough to make contacts with tubulin in the complex structures (as determined with UCSF-Chimera: find contacts utility) are highlighted in bright green. From top to bottom the kinesin sequences in each panel correspond to: KLP10A in the $NMMT_{apo}$, $NMMT_{AMP\text{-}PNP}$ and $CTMMT_{AMP\text{-}PNP}$ complexes, Kinesin-1-tubulin complexes in the apo form (4LNU) and in the presence of $ADPAlF_4$ (4HNA). Note that tubulin in the two kinesin-1 tubulin complex structures is in the curved form, so it is possible that some of the kinesin-1 mapped interactions may be different when binding to the microtubule lattice.

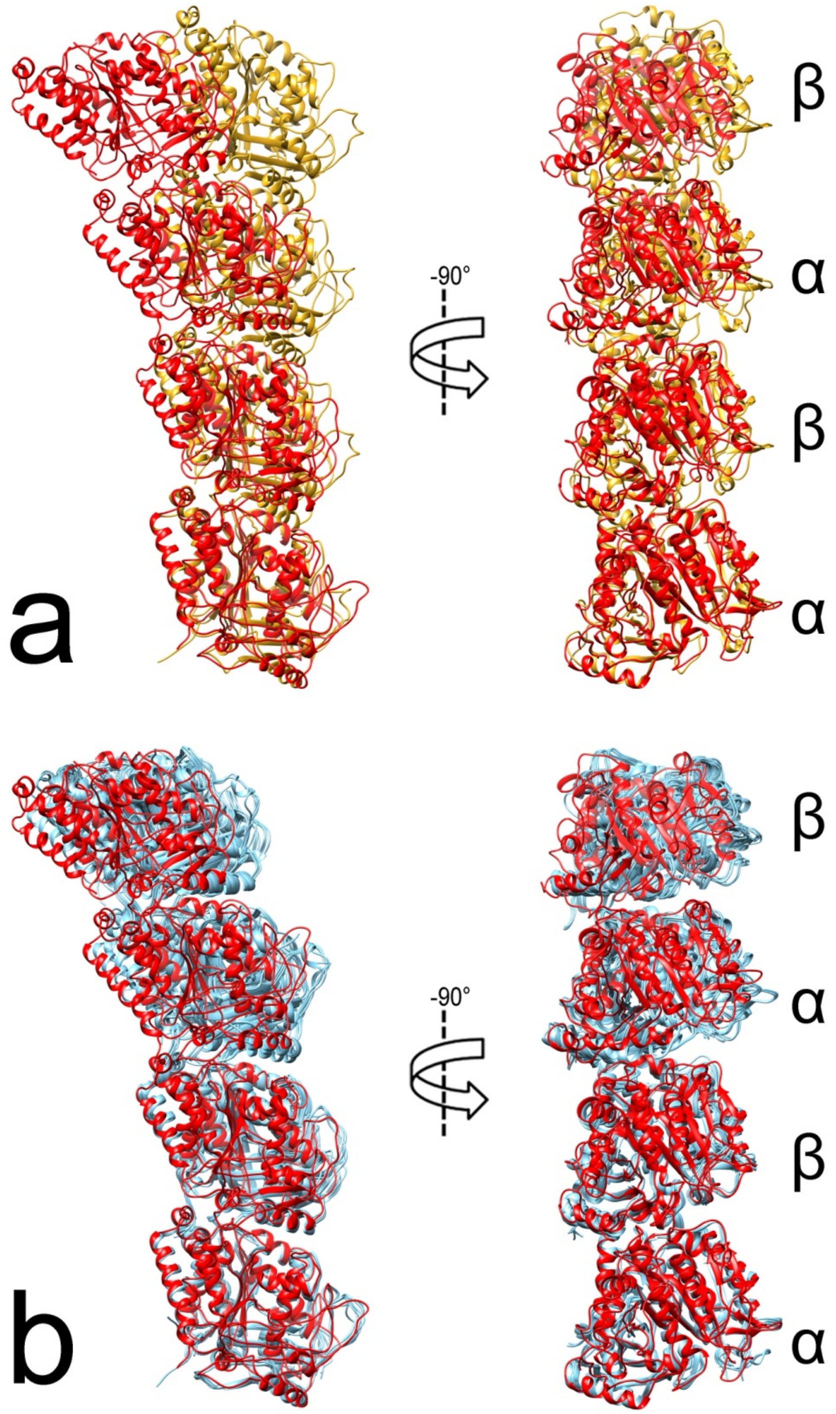

Supplementary Fig. 6 Comparison between tubulin protofilament structures. a Tubulin protofilament in the $CTMMT_{AMP-PNP}$ complex (red) vs. the straight tubulin in the microtubule of the same complex (yellow). **b** Tubulin protofilament in the $CTMMT_{AMP-PNP}$ complex (red) vs. several superimposed crystal structures of curved tubulin protofilaments (cyan, PDB accession codes: 1SA0, 3RYF, 3RYH, 3RYI, 3RYC, 4I4T, 4I55, 4IHJ). All structures aligned by their bottom α-tubulin structure.

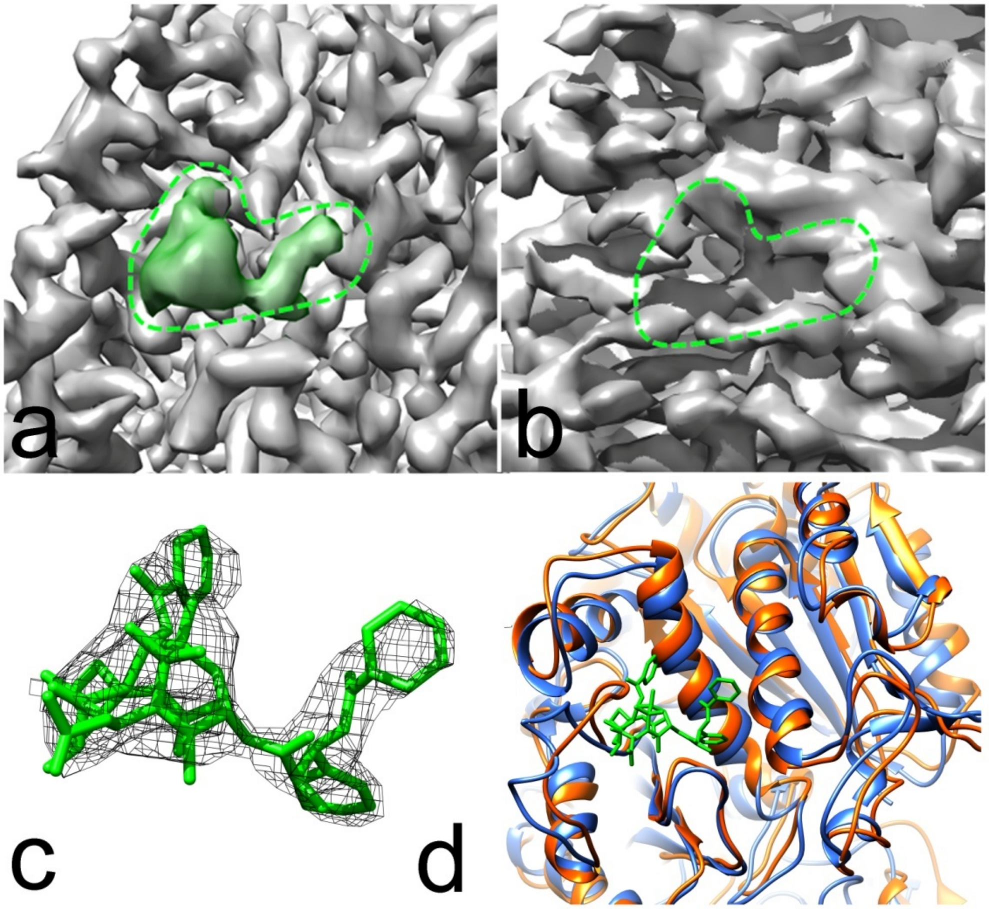

Supplementary Fig. 7 Paclitaxel binding site. a Cryo-EM density (iso-surface representation) near the paclitaxel (Taxol®) binding site in the β-tubulin subunit of the microtubule in the $CTMMT_{AMP\text{-}PNP}$ complex. The density corresponding to the paclitaxel molecule is colored green. The paclitaxel molecule with associate density is showed in (c). **b** Cryo-EM density near the paclitaxel binding site in the β-tubulin subunit of the curved tubulin (CT) in the $CTMMT_{AMP\text{-}PNP}$ complex. Despite the maps having different resolution in this area (Supplementary Figs. 2 and 3) a distinct cavity can be observed in (b) in the place that is occupied by paclitaxel in (a) (dotted green outline). **c** Paclitaxel molecule (green) and associated cryo-EM density (dark mesh) in the β-tubulin subunit of the microtubule in the $CTMMT_{AMP\text{-}PNP}$ complex. **d** Comparison of the two β-tubulin models in the $CTMMT_{AMP\text{-}PNP}$ complex. The microtubule β-tubulin is shown in blue with paclitaxel molecule in green. The curved tubulin protofilament β-tubulin is shown in orange.

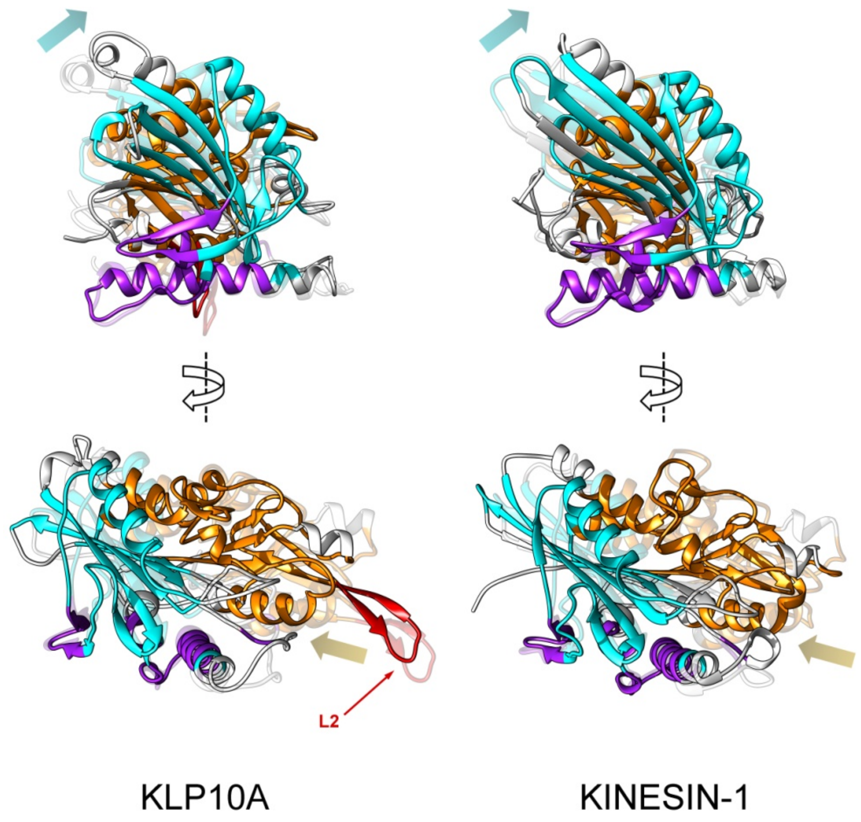

Supplementary Fig. 8 Kinesin motor domain movements associated with nucleotide pocket closure. Confomational changes in kinesin-1 associated with binding of ATP analogues have been described as relative movements between three sub-domains (Cao et al., 2014), a 'P-loop' sub-domain (colored orange), a 'Switch I/II' sub-domain (colored cyan) and a 'tubulin binding' sub-domain (colored magenta). The left panel shows the KLP10A motor-domain of the $CTMMT_{AMP\text{-}PNP}$ complex (100% opacity) superimposed with the one of the $NMMT_{apo}$ complex (30% opacity). The right panel shows the kinesin-1 motor-domain of the KIF5B-$ADPALF_4$-tubulin complex (4HNA, 100% opacity) superimposed with the one of the KIF5B-apo-tubulin complex (4LNU, 30% opacity). Sequence aligned regions of KLP10 and KIF5B are colored the same. Superimposed structures are aligned by their 'tubulin binding' sub-domains. Note that similar sub-domains movements occur in KLP10A and kinesin-1 going from the apo (and open nucleotide pocket) form to the ATP-analogue-bound (and closed nucleotide pocket) form. This includes a rotation of H4 relative to the rest of the motor domain, first detected in medium resolution cryo-EM structures of kinesin microtubule complexes[38]. H4 is part of the 'tubulin binding' sub-domain colored magenta in this Figure. The rotation of H4 relative to the rest of the KLP10A motor domain can also be seen clearly in Figs. 2(d)-(f). In the case of KLP10A the kinesin-13 family-specific loop-2 (red), which is connected to the 'P-loop sub-domain', moves relative to the 'tubulin-binding' sub-domain. This movement induces a tubulin conformational change because loop-2 stays bound to tubulin (Figs. 2 and 3, Supplemental Fig. 9).

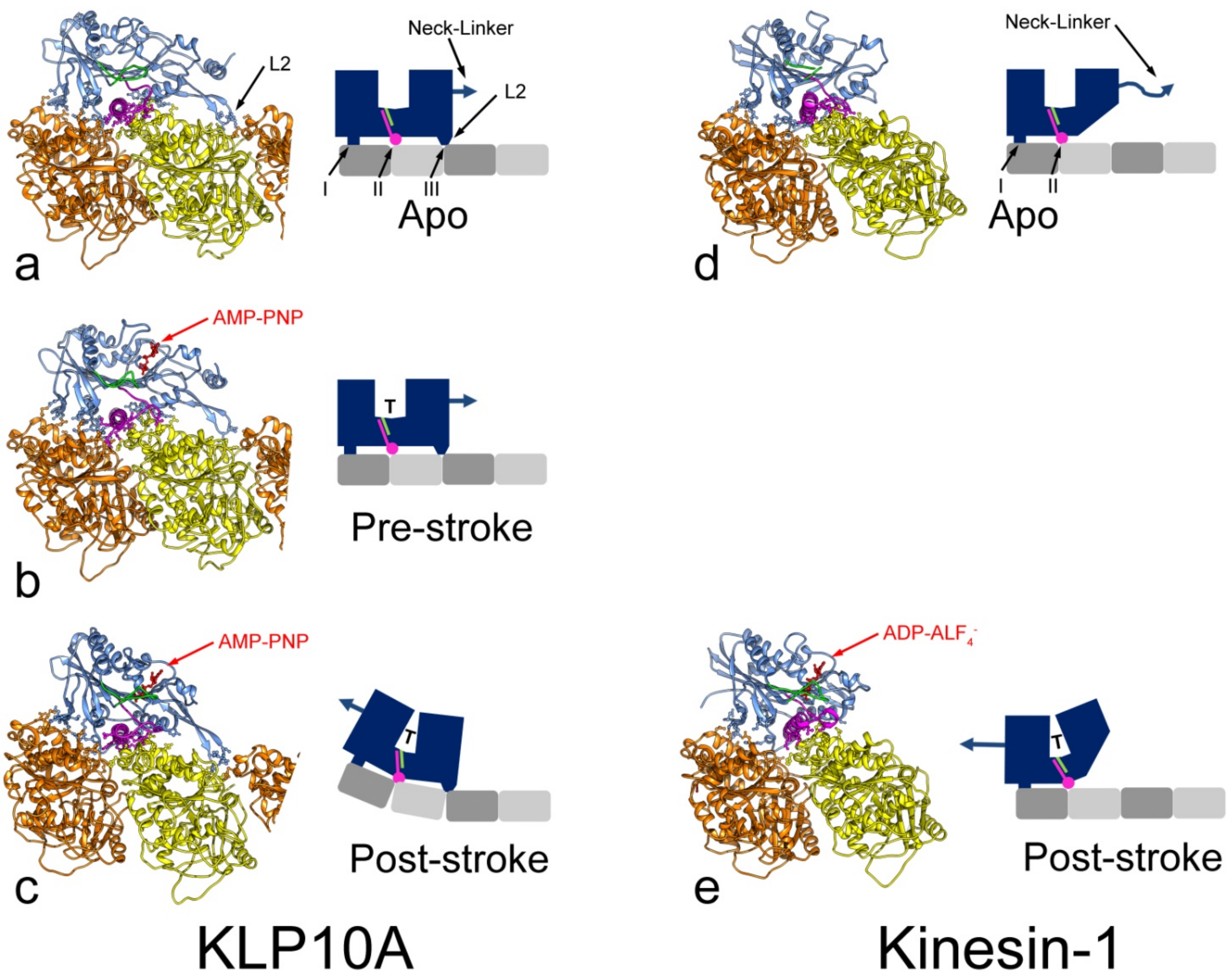

Supplementary Fig. 9. Conserved kinesin ATPase related conformational changes adapted for microtubule depolymerization or unidirectional movement. Atomic models (left) and cartoon interpretation of the structures (right). **a** KLP10A $NMMT_{apo}$ complex. **b** KLP10A $NMMT_{AMP\text{-}PNP}$ complex. **c** KLP10A $CTMMT_{AMP\text{-}PNP}$ complex. **d** Kinesin-1 (KIF5B) tubulin complex in the apo form (4LNU). **(e)** Kinesin-1 (KIF5B) tubulin complex with $ADPAlF_4$ (4HNA).
