# Supplementary material for: Cryo-EM reveals the structural basis of microtubule depolymerization by kinesin-13s
Source: Nat Commun. 2018 Apr 25;9:1662. doi: 10.1038/s41467-018-04044-8 (PMC5916938; doi:10.1038/s41467-018-04044-8)
Supplement: Supplementary file 3 — Description of Additional Supplementary Info [file 41467_2018_4044_MOESM3_ESM.pdf]

Description of Additional Supplementary Files

File Name: Supplementary Movie 1
Description: Morph between NMMTapo and CTMMTAMP-PNP models with structures aligned on the KLP10A P-loop. KLP10A in blue; KLP10A P-loop in pink; KLP10A SW1 in green; KLP10A SW2-H4 in magenta, α-tubulin in light gray, β-tubulin in dark gray, AMP-PNP in red.

File Name: Supplementary Movie 2
Description: Morph between NMMTAMP-PNP and CTMMTAMP-PNP models with structures aligned on the KLP10A P-loop. KLP10A in blue; KLP10A P-loop in pink; KLP10A SW1 in green; KLP10A SW2-H4 in magenta, α-tubulin in light gray, β-tubulin in dark gray, AMP-PNP in red.

File Name: Supplementary Movie 3
Description: Morph between NMMTapo and NMMTAMP-PNP models with structures aligned on the KLP10A P-loop. KLP10A in blue; KLP10A P-loop in pink; KLP10A SW1 in green; KLP10A SW2-H4 in magenta, α-tubulin in light gray, β-tubulin in dark gray, AMP-PNP in red.

File Name: Supplementary Movie 4
Description: KLP10A-Tubulin conformational changes seen from the protofilament perspective. Morph between NMMTapo, NMMTAMP-PNP and CTMMTAMPPNP models with structures aligned on the left-most β-tubulin subunit. KLP10A in blue; KLP10A P-loop in pink; KLP10A SW1 in green; KLP10A SW2-H4 in magenta, α- tubulin in light gray, β-tubulin in dark gray, AMP-PNP in red. KLP10A neck domain not shown.

File Name: Supplementary Movie 5.
Description: KLP10A-Tubulin conformational changes seen from the KLP10A nucleotide binding pocket perspective. Morph between NMMTapo, NMMTAMP-PNP and CTMMTAMP-PNP models with structures aligned on the P-loop of leftmost KLP10A motor domain; KLP10A in blue; KLP10A P-loop in pink; KLP10A SW1 in green; KLP10A SW2-H4 in magenta, α-tubulin in light gray, β-tubulin in dark gray, AMP-PNP in red. KLP10A neck domain not shown.
