# Supplementary material for: Cryo-EM reveals the structural basis of microtubule depolymerization by kinesin-13s
Source: Nat Commun. 2018 Apr 25;9:1662. doi: 10.1038/s41467-018-04044-8 (PMC5916938; doi:10.1038/s41467-018-04044-8)
Supplement: Supplementary file 2 — Peer Review Report [file 41467_2018_4044_MOESM2_ESM.pdf]

Reviewers' comments:

Reviewer #1 (Remarks to the Author):

The paper by Benoit et al aims to address central questions concerning the mechanism of kinesin-13 motors. While substantial progress has been made in the understanding of the molecular mechanism of kinesin stepping towards microtubule plus ends, especially of kinesin-1, still relatively little is known about the kinesin-13 microtubule depolymerization mechanism. Benoit and colleagues present a beautiful set of high resolution cryo-EM structures of kinesin-13 monomer constructs of the Drosophila melanogaster kinesin-13 KLP10A. The motor is captured in different nucleotide states, and bound both to the microtubule lattice and curved tubulin protofilaments that likely represent intermediates in the depolymerization process.

Altogether, this work puts the authors in a strong position to shed important light on the kinesin-13 mechanism. I am very enthusiastic about these data. However, the current version of the manuscript does not provide many mechanistic insights. While the Introduction is a succinct and lucid account of the current literature and the Methods text is comprehensive and clear, the Results & Discussion text, together with the figures, would benefit from a major overhaul and the mechanistic analysis should be revisited. If these points could be addressed, I anticipate the paper would be highly significant for the field.

1. Central mechanistic questions are how the kinesin-13 ATPase is preferentially stimulated by curved tubulin compared to straight lattice tubulin, and what conformational changes occur in kinesin and tubulin as a result. However, the text tends towards a description of the differences between the determined structures – e.g. there are differences in the central b-sheet, in loop5 (vs other kinesins), in the microtubule binding surface - rather than a mechanistic dissection of these differences.

Substantial progress has been made in the structural elucidation of the kinesin-1 mechanism. Comparison of these new kinesin-13 structures with the high resolution apo and AMPPNP tubulin-bound structures of kinesin-1 motor domain would seem an obvious place to start in a mechanistic analysis. For example, are there subdomain movements within the kinesin-13 motor domain as are seen in other plus end directed kinesins? Such a comparison would also allow the authors to support their statement that the kinesin-13 apo state fits the conformation of lattice tubulin. The dogma in the field (e.g. Ogawa et al, 2004) has been that the tubulin-binding surface of kinesin-13 motors have a fundamentally different shape than transport kinesins, contributing to their depolymerizing properties. Is that true and/or does it change in different nucleotide states?

The authors' mechanistic interpretations are determined by how they perform their structural alignment: for example an alignment on beta-tubulin is shown in Fig3B, while the kinesin-13 is allowed to move at the motor-tubulin interface; since the tubulin is the substrate of the motor it is not obvious that this makes sense. Conversely, the movies use alignment on the motor P-loop, but the potentially very striking structural effects that this comparison allows are not described in the text. What is the logic here?

2. The first paragraph of the results would benefit from the inclusion of a more thorough recap of the constructs used in the work, a more thorough rationale for the structures solved and a description for the logic of the order in which they are presented. Most of the high resolution analysis is performed on the longer NM construct but in the 3D structures on the microtubule lattice, most of the neck is not visible, so 2D analysis of curved tubulin rings is undertaken to obtain more insight. This is fine but is not very clearly explained.

3. The conceptual parallel between movements of L2 and "powerstroke"-like conformational changes in other motors is unconvincing – the only discernible link is that conformational changes are seen in response to nucleotide. Movements of the neck in response to nucleotide would be a

more obvious candidate for this analogy, but unfortunately, the current set of structures do not shed light on this.

4. The authors recapitulate at higher resolution the earlier observation (Mulder et al, 2009) that neck-containing kinesin-13 constructs bind alternate tubulin dimers in curved tubulin oligomers. The higher resolution of the current data allows direct visualisation of density attributed to the neck region trailing at the minus end of motor domain. Given the heterogeneity of these data (illustrated in Fig4B) and that these are 2D rather than 3D data, the authors cannot be sure that they have visualised an extended alpha-helix, and the text should accordingly be more caveated.

5. While the overall configuration of tubulin in the different structures are described, no comparison is provided with previously solved structures of tubulin. This would be particularly interesting for the curved protofilaments bound by KLP10A. Is there a fundamental difference in curvature in the presence of bound kinesin-13? The authors previously proposed an alternative, distorted conformation of this curved tubulin based on lower resolution reconstructions of KLP10A-tubulin complexes (Asenjo et al, 2013). How does the conformation of tubulin they now visualise compare with that?

Minor points:
- 1st line of results/discussion: KLP10A is said to form stable complexes – is this the full length protein or truncated constructs? The text should be clarified.
- would be useful to state the distinct resolution of microtubule and kinesin parts of each 3D reconstruction in the main text as well in the supplementary figure so readers' expectations are appropriately set
- p5, line 15 typo: “specie”
- p9, line 13 typo: “trough”
- through the methods, volumes are expressed in micro-liters, microliters and µL – these need to be consistent.

Reviewer #2 (Remarks to the Author):

This paper describes important new data on the structure and transitions of a Kinesin-13 motor that removes tubulin dimer from microtubules (MTs). Cryo-EM was used to obtain reconstructions of motor-MT complexes in three nucleotide states that represent important stages of the binding/hydrolysis cycle of the motor. With resolution sufficient to build reliable atomic models of the complex, the authors demonstrate convincingly that the tubulin and kinesin undergo complementary changes in conformation that lead to a tubulin dimer being incompatible with the microtubule lattice. Modifications to the data processing protocols that enabled these results are reasonably well described. Overall, the work is technically impressive, and the results are a significant step towards understanding the mechanism of MT depolymerization. This process can now be understood at around the same level as the mechanism of regular kinesin stepping along MTs, and perhaps even better. Details on the forces that drive the conformational changes may still need to be explained, but this work gives a nice empirical model of what happens through the mechanical cycle.

I have almost no complaints about the paper, but would offer one thought on the mechanisms investigated:

P. 9: the authors state “the structure implies that KLP10A is not catalytic when bound to the microtubule-lattice trough the putative kinesin-tubulin binding site”. This is certainly consistent with their data, but begs the question of what comes first to activate catalysis – closure of the pocket (presumed transiently) or tubulin curvature. It seems that the model suggested is that binding of the ATP-loaded kinesin puts stress on the protofilaments to make them curve. It might be a significant addition supporting this model if it were easy to show that AMP-PNP-loaded

KLP10A promotes some level of MT depolymerization. Perhaps there are already enough images of MT ends to get some insight on this point, although the presence of paclitaxel could confound interpretation.

Otherwise, just a few minor typographical points:

p. 9 ‘trough’ should probably be ‘through’
p. 13 ‘on a solution of microtubule’ should probably be ‘for a solution of tubulin’
p. 15, line 1 into >> onto
2 times – ‘where’ >> were
p. 16, step 2: R of L >> R or L
‘segment corresponding’ >> segments corresponding’
p. 21 ‘two tubulin heterodimers has one kinesin’ >> ’two tubulin heterodimers has two kinesin’

Reviewer #3 (Remarks to the Author):

Benoit et al., Cryo-EM Reveals the Structural Basis ...

This paper is a structural and functional study into the mechanism and microtubule interaction of kinesin-13, a plus-end directed microtubule-based molecular motor. These kinesins are special in their way as they do not seem to be very motile, but act as a microtubule depolymerizer at the plus-ends of tubulin protofilaments (although the apparent lack of motility may only seem to be the case mostly because they accumulate at the minus-ends where they continue hydrolyzing ATP, causing microtubule protofilaments to fall apart). This group has already published several interesting structural studies on that topic, and comprises a high level of expertise on cryo-electron microscopy 3-D analysis of microtubule-kinesin complexes. Here they deliver a high-quality, high-resolution investigation into the structural details of how these motors achieve their depolymerizing task. This is a logical continuation of their work and now achieves near atomic resolution (the authors claim ~4Å). This enough details that allow for a very precise correlative molecular interpretation with the kinesin X-ray structures at hand.

Methods are very precisely explained and highlight the excellent quality of the work presented here. It allows the reviewer to appreciate the effort that has been taken here. It is oblviously a great strike of luck that the protofilament-wrapping configuration (Fig. 1B) made it into a stable helical complex. With this complex at hand it seems that the bending conformation is very well simulated and allows for significant interpretations between regular kinesin-protofilament binding and the one that seems to take microtubules apart. Otherwise these complexes would have been very hard to get to high resolution as it was achieved here.

To me, doing related work in this field, this paper is of high relevance to the community, superbly executed, and therefore ready for immediate publication. The structural details observed between the three different states that were investigated are simply stunning and should be made public as soon as possible. The proposed model is plausible and will provide an excellent basis for any types of ongoing structural studies into kinesin-microtubule interactions in general. I have nothing more to add, and no revisions are necessary, in my opinion.

Response to reviewers' comments

We thank the reviewers for their critical assessment of our manuscript. We appreciate that all three reviewers considered the data presented to be of high quality and to provide important new insights into the mechanism of kinesin-13 induced microtubule depolymerization. Nevertheless, there were also several comments, mostly related to the discussion of our data. To address these comments we have made extensive modification to the text, modified existing figures, added two new main figures and three new supplementary figures. Following is a list of the major changes and a point by point response to the reviewer comments.

Major Changes:

Results and Discussion:

-New sections titled:
'The KLP10A-tubulin interface'
'Comparison with other kinesins and mechanism of microtubule depolymerization'

-The text in the previous section with the title heading 'A pre-power stroke configuration', was modified and incorporated under the new section 'Comparison with other kinesins and mechanism of microtubule depolymerization'.

-The description of the structures now follows the order $NMMT_{apo}$ -> $NMMT_{AMPPNP}$ -> $CTMMT_{AMPPNP.}$ Changes were made in the text and related figures accordingly.

Figures & videos:

-Panels g, h, i added to Fig. 2.
-New Fig. 3.
-New Fig. 4. (modified version of previous Fig. 3)
-Added 2 panels to Fig. 5 (previous Fig. 4).
-New Fig. 6.
-Modified Fig. 7 (previous Fig. 5).

- Supp Fig. 1: added panel e.
- New Supp. Fig. 5. Replaces previous Supp. Fig 5.
- New Supp. Fig. 6
- New Supp. Fig. 8.
- New Supp.. Fig 9.
- New Supp. Movie 5.

Point to point responses (reviewer comments in *italic*):

Reviewer 1:

However, the current version of the manuscript does not provide many mechanistic insights. While the Introduction is a succinct and lucid account of the current literature and the Methods text is comprehensive and clear, the Results & Discussion text, together with the figures, would benefit from a major overhaul and the mechanistic analysis should be revisited. If these points could be addressed, I anticipate the paper would be highly significant for the field.

Making the connection between structure and mechanism is the main goal of our manuscript so we took this comment to heart and have made extensive modifications to the text and illustrations to help making this connection. In particular:

The last part of the results and discussion section now under the new heading 'Comparison with other kinesins and mechanism of microtubule depolymerization' was rewritten to synthesize the structural observations into the proposed mechanism of microtubule depolymerization.

The new Fig. 3 was added to better make the connection between the structural changes occurring in the KLP10A nucleotide binding pocket (closure) and other confomational changes within the motor-domain leading to a change in the shape of the tubulin interface and tubulin bending. In the new supplementary Figs. 8 and 9 we compare the observed sub-domain movement in KLP10A with the ones in kinesin-1.

The previous Fig. 5 (now Fig. 7) was modified to better make the connection between the structural observations and the proposed mechanism of microtubule depolymerization, including a comparison with the kinesin-1 translocation mechanism.

1. kinesin-1 mechanism. Comparison of these new kinesin-13 structures with the high resolution apo and AMPPNP tubulin-bound structures of kinesin-1 motor domain would seem an obvious place to start in a mechanistic analysis.

We have now made a more thorough comparison with kinesin-1 tubulin complexes and other kinesin structures in a new section under the heading: "Comparison with other kinesins and mechanism of microtubule depolymerization".

Three new supplementary figures (Supp Figs. 5, 8, 9) were added where we compare our structures with the high resolution kinesin-1-tubulin structures indicated.

For example, are there subdomain movements within the kinesin-13 motor domain as are seen in other plus end directed kinesins?

Yes there are sub-domain motions that are common with other kinesin proteins. Basically, our model is that the sub-domain motions associated with closure of the nucleotide binding pocket are similar in

kinesin-13s and kinesin-1s (and probably across kinesins). In the case of kinesin-13s the family conserved loop-2 is used to couple these kinesin motor sub-domain motions to tubulin curvature. This is shown and discussed in the Results and Discussion section 'Comparison with other kinesins and mechanism of microtubule depolymerization, in the new Figure 7 and in the new supplemental Figs. 8 and 9.

The dogma in the field (e.g. Ogawa et al, 2004) has been that the tubulin-binding surface of kinesin-13 motors have a fundamentally different shape than transport kinesins, contributing to their depolymerizing properties. Is that true? and/or does it change in different nucleotide states?

We have argued in the past (e.g. Asenjo et al 2013) that the unique characteristics of kinesin13s cannot be simply explained by its motor domain surface being more convex (to adapt the more concave curved tubulin surface) as originally proposed by Ogawa et al.

Our new data we believe further refute this early hypothesis. First, excluding the additional kinesin-13 loop-2 (L2), the tubulin interacting surface of KLP10A and kinesin-1 are very similar (not more or less convex) and there are many conserved residues in these areas in KLP10A and kinesin-1 interacting with tubulin (Supp Fig. 5). Second, as shown now in this paper the position of L2 relative to the other binding areas is different depending whether the motor is bound to straight or curved tubulin. The data shows that the shape of the kinesin-13-tubulin interface and the motor domain nucleotide site are coupled by the additional tubulin interactions of L2.

The authors' mechanistic interpretations are determined by how they perform their structural alignment:

The given mechanistic interpretations are independent of how the structures are aligned. We choose two types of alignments or references to highlight movement of one domain or sub-domain relative to another.

..for example an alignment on beta-tubulin is shown in Fig3B, while the kinesin-13 is allowed to move at the motor-tubulin interface; since the tubulin is the substrate of the motor it is not obvious that this makes sense...

When aligning on β-tubulin we are highlighting how conformational changes in the bound KLP10A result in the movement of α-tubulin and the rest of the protofilament relative to this particular β-tubulin, Note that the interface between the stationary β-tubulin and kinesin, what we refer as area I, remains in the same place while other areas of the interface (referred as area III) move causing tubulin bending. i.e. Area III of the interface moves relative to areas I and II and α-tubulin moves relative to β-tubulin.

With this alignment we are highlighting how the conformational changes in the motor domain are coupled to tubulin bending and protofilament peeling from the microtubule.

Conversely, the movies use alignment on the motor P-loop, but the potentially very striking structural effects that this comparison allows are not described in the text. What is the logic here?

When aligning on the P-loop we are highlighting how the switch domains move closer to the P-loop as the nucleotide binding pocket closes. This is what we refer as closing of the nucleotide binding pocket through the text and figures. We added new panels to Figure 2 (g-i) and a new Fig. 3 to better make this point.

Regardless of how the structures are aligned the mechanism is the same. The protofilament bends when the KLP10A nucleotide pocket of the attached motor domains closes. With the two types of alignments we tried to convey the same process from the microtubule perspective or form the nucleotide binding pocket perspective. To further illustrate this point we added a new supplementary movie 5. It is a similar morph as the one shown in supp. movie 4 but observed from the reference point of view of the nucleotide binding pocket of one of the two KLP10A motor domains.

2. The first paragraph of the results would benefit from the inclusion of a more thorough recap of the constructs used in the work, a more thorough rationale for the structures solved and a description for the logic of the order in which they are presented.

We added some text to this section accordingly. We also changed the order in which we present the structures (now NMMT(apo) -> NMMT(AMPPNP) -> CTMMT(AMPPNP)) hopefully to make the reading easier.

Most of the high resolution analysis is performed on the longer NM construct but in the 3D structures on the microtubule lattice, most of the neck is not visible, so 2D analysis of curved tubulin rings is undertaken to obtain more insight. This is fine but is not very clearly explained.

We expanded the text under the heading 'Conformation and Position of the KLP10A-Neck-Domain' and added a new panel to Fig. 5 to better explain this part.

3. The conceptual parallel between movements of L2 and "powerstroke"-like conformational changes in other motors is unconvincing – the only discernible link is that conformational changes are seen in response to nucleotide. Movements of the neck in response to nucleotide would be a more obvious candidate for this analogy, but unfortunately, the current set of structures do not shed light on this.

We borrowed the term power-stroke in its more general use to highlight the analogy with other motor proteins. E.g. "A power stroke is large, rapid structural change in a protein that can be used to do mechanical work... The idea is that the protein has two different structural states, and that the binding or unbinding of a small molecule — or a chemical change in a bound molecule such as the hydrolysis of ATP — switches the protein from being primarily in structural state 1 to being primarily in structural state 2" (Howard J. Protein Power Strokes. 16, pR517–R519 2006).

However, we agree that it can also be taken literally as the rotation of an elongated lever-arm structure as was first proposed for myosin-II. We still believe that making the analogy with the key conformational changes of other motor proteins is important, but to avoid confusion we now use the terms pre-stroke and post-stroke in Fig. 7 and Supplementary Fig. 9.

4. The authors recapitulate at higher resolution the earlier observation (Mulder et al, 2009) that neck-containing kinesin-13 constructs bind alternate tubulin dimers in curved tubulin oligomers. The higher resolution of the current data allows direct visualisation of density attributed to the neck region trailing at the minus end of motor domain. Given the heterogeneity of these data (illustrated in Fig4B) and that these are 2D rather than 3D data, the authors cannot be sure that they have visualised an extended alpha-helix, and the text should accordingly be more caveated.

We added the caveat "...Although 3D data for this part of the complex would be required to fully determine its structure..."

5. While the overall configuration of tubulin in the different structures are described, no comparison is provided with previously solved structures of tubulin.

We now included this comparison in the text under the heading 'Tubulin conformational changes' and in a new supplementary figure (Supplementary Fig. 6).

Is there a fundamental difference in curvature in the presence of bound kinesin-13?
Yes, kinesin-13 induces higher curvature. Discussed now in text and shown in Fig. 5C and new supp. fig. 6.

The authors previously proposed an alternative, distorted conformation of this curved tubulin based on lower resolution reconstructions of KLP10A-tubulin complexes (Asenjo et al, 2013). How does the conformation of tubulin they now visualise compare with that?

There is much less shear in the present model derived from the higher resolution data. This is now stated in the text under the heading 'Tubulin conformational changes' and illustrated in the new Supplementary Fig. 6.

Minor points:
- 1st line of results/discussion: KLP10A is said to form stable complexes – is this the full length protein or truncated constructs? The text should be clarified.

This is a general statement indicating that as far as we know any construct that includes the motor domain (from motor domain only to near full length) can form these complexes. Further below we specify the particular constructs used in this work. We modified the text to clarify this point: Now: "... Examples of these complexes obtained with the KLP10A constructs used in this work are shown in Supplementary Figs. 1b, d, e."

- would be useful to state the distinct resolution of microtubule and kinesin parts of each 3D reconstruction in the main text as well in the supplementary figure so readers' expectations are appropriately set

Done.

- p5, line 15 typo: "specie"

Done.

- p9, line 13 typo: "trough"

Done.

- through the methods, volumes are expressed in micro-liters, microliters and µL – these need to be consistent.
Done

Reviewer 2:
P. 9: the authors state "the structure implies that KLP10A is not catalytic when bound to the microtubule-lattice trough the putative kinesin-tubulin binding site". This is certainly consistent with their data, but begs the question of what comes first to activate catalysis – closure of the pocket (presumed transiently) or tubulin curvature. It seems that the model suggested is that binding of the ATP-loaded kinesin puts stress on the protofilaments to make them curve. It might be a significant addition supporting this model if it were easy to show that AMP-PNP-loaded KLP10A promotes some level of MT depolymerization. Perhaps there are already enough images of MT ends to get some insight on this point, although the presence of paclitaxel could confound interpretation.

Yes we believe that an ATP loaded kinesin put stress on the protofilament to bend it. There is evidence that AMP-PNP loaded KLP10A does promote MT depolymerization. The longer the incubation of AMPPNP-KLP10A with microtubules the more curved protofilament complexes (both isolated and wrapped along the remaining MTs) are observed. This is consistent with some earlier observations of MT depolymerization in the presence of AMP-PNP and another kinesin-13 (Moores et al., 2002, in the references). We and others have also reported the observation of curved protofilaments at the end of microtubules in the presence of ATP and AMPPNP. We have now added more examples of this from the samples used in this manuscript (Supp. Fig. 1e). This together with the fact that the end of microtubules, without adding kinesin-13 or inducing depolymerization by other means, tend to be blunt does indicate that kinesin-13s actively bend protofilaments. Having said that, the fact that KLP10A and other kinesin-13s in the presence of AMPPNP also bind well to unpolymerized tubulin (presumably curved tubulin) shows that an alternative route to the closed-pocket catalytic state is for K-ATP to bind to an already curved protofilament and perhaps then inducing further curvature as our data suggests.

p. 9 'trough' should probably be 'through'
Corrected.

p. 13 'on a solution of microtubule' should probably be 'for a solution of tubulin'
Corrected

p. 15, line 1 into >> onto
We didn't find 'into' in the first line of page 15 but corrected several instances of 'into' through the text.

2 times – 'where' >> were
Several instances of incorrect where/were usage were fixed.

p. 16, step 2: R of L >> R or L
Fixed

'segment corresponding' >> segments corresponding'
Fixed.

p. 21 'two tubulin heterodimers has one kinesin' >> 'two tubulin heterodimers has two kinesin'
This sentence was reworded as:
One in which there is a kinesin density associated with each tubulin heterodimer (two kinesins and two tubulin heterodimers) and another with one kinesin per two tubulin heterodimers.

REVIEWERS' COMMENTS:

Reviewer #1 (Remarks to the Author):

The authors have done a very nice job of the rewrite of this interesting manuscript, with a number of highly significant observations for the field now clearly described.

Newly included in the rewrite (p11), the comparison with kinesin-1 throws up the very striking observation of a neck-linker-like docking of the C-terminus of kinesin-13. Although not a major point of the current study, this intriguing and provocative observation deserves a little more elaboration (maybe a sentence each) concerning: i) whether the kinesin-13 sequence has any similarity to the classical neck-linker sequence in motile kinesins; ii) the similar phenomenon observed in minus end directed kinesin-14s; iii) any data about the functional evidence for the role of the C-terminus in kinesin-13 function.

Minor
- Please check spelling/grammar in 1st sentence of 2nd paragraph of introduction
- Please check errors in 1st sentence, 2nd para, p12: “Based ON our results…..loop-2 with the tubulin INTER-dimer interface…”
- Legend to Figure 7: refers to Stub/CTub but these labels are not included in the figure itself – I don’t think they are needed
